# Nanoparticulation of Prodrug into Medicines for Cancer Therapy

**DOI:** 10.1002/advs.202101454

**Published:** 2021-07-29

**Authors:** Yuezhou Zhang, Huaguang Cui, Ruiqi Zhang, Hongbo Zhang, Wei Huang

**Affiliations:** ^1^ Frontiers Science Center for Flexible Electronics (FSCFE) Xi'an Institute of Flexible Electronics (IFE) and Xi'an Institute of Biomedical Materials & Engineering (IBME) Northwestern Polytechnical University 127 West Youyi Road Xi'an 710072 China; ^2^ Ningbo Institute of Northwestern Polytechnical University 218 Qingyi Road Ningbo 315103 China; ^3^ Pharmaceutical Sciences Laboratory Åbo Akademi University Turku FI‐00520 Finland; ^4^ Turku Bioscience Centre University of Turku and Åbo Akademi University Turku FI‐00520 Finland

**Keywords:** cancer therapy, endogenous/exogenous stimuli, nanoprodrug, on‐demand release, polyprodrug

## Abstract

This article provides a broad spectrum about the nanoprodrug fabrication advances co‐driven by prodrug and nanotechnology development to potentiate cancer treatment. The nanoprodrug inherits the features of both prodrug concept and nanomedicine know‐how, attempts to solve underexploited challenge in cancer treatment cooperatively. Prodrugs can release bioactive drugs on‐demand at specific sites to reduce systemic toxicity, this is done by using the special properties of the tumor microenvironment, such as pH value, glutathione concentration, and specific overexpressed enzymes; or by using exogenous stimulation, such as light, heat, and ultrasound. The nanotechnology, manipulating the matter within nanoscale, has high relevance to certain biological conditions, and has been widely utilized in cancer therapy. Together, the marriage of prodrug strategy which shield the side effects of parent drug and nanotechnology with pinpoint delivery capability has conceived highly camouflaged Trojan horse to maneuver cancerous threats.

## Graphical abstract

Prodrugs release bioactives on‐demand at diseased sites to reduce systemic toxicity of parent drugs; the nanotechnology formulate the prodrug into nanosized. Their marriage boosts an emerging and effective solution to cancerous implications and beyond. The nanoprodrug inherits the features of both prodrug concept and nanomedicine expertise, attempting to address unmet needs in cancer treatment cooperatively.

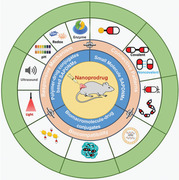

## 1 Introduction

Prodrugs are chemical entities with few or no pharmacological activity at all. After being taken, they are transformed into the active parent drug by variety of stimuli. Prodrugs have been discovered by chance or planned purposely. Those attempts are tried to address drug development barriers that confine formulation choices, lead to unwanted features, and off‐targeting outcomes.^[^ ^1^ ^]^ To meliorate the bioavailability of poorly water‐soluble drug, the native drug is often modified into a hydrophilic version.^[^ ^2^, ^3^ ^]^ This strategy has been utilized to “redeem” water insoluble drug candidates.^[^ ^4^ ^]^ More than 30 prodrug products have been commercialized during past decade.^[^ ^1^ ^]^ Depending upon how the prodrug was converted into the final active drug, they are categorized into two major types: type I prodrugs are intracellularly activated and type II prodrugs are extracellularly activated, particularly in digestive fluids or blood.

Nanotechnology attempt to manipulate/understand matter at the scale of 1 to 100 nm, in which unique properties imply novel applications.^[^ ^5^ ^]^ Translational medicine is defined as “an interdisciplinary branch of the biomedical field supported by benchside, bedside and community”,^[^ ^6^ ^]^ which tends to fill the gap between “what we know” and “what we practice.” Nanotechnologies for translational medicine, or nanomedicine focus on the use of precisely engineered nanomaterials to develop novel diagnostic and therapeutic entities.^[^ ^7^ ^]^ The progress toward some of goals has been discussed and the advances which likely occur during the next decade are also predicted.^[^ ^8^ ^]^ Nanoparticulation represents one of the most promising strategies for nanotechnology applications.^[^ ^9^ ^]^ Unlike the “top‐down” approaches, a “bottom‐up” nanoparticulation favors the formation of nanoscale architectures fueled by noncovalent self‐assembly. Self‐assembly of nanoparticles (NPs) has been defined as the function of components spontaneously bridged into ordered structures either through direct interparticle forces, or indirectly using a template or an external field and is characterized by a minimum free energy in the system.^[^ ^10^ ^]^ A merit of self‐assembled system is molecular cooperativity where the get‐together behavior differs as a whole from the sum of individuals. The application of how to integrate molecular cooperativity to fabricate self‐assembled nanomedicine with enhanced therapeutic efficacy has been recently discussed.^[^ ^11^ ^]^ Two methods of prodrug nanoformulations in practice have been implemented. First one is to chemically conjugate bio/synthetic polymer with drugs then allow the conjugates to assemble into nanostructures spontaneously. Another one is to couple small molecules with other low molecular weight drug to obtain a conjugate which self‐assembles into NPs in aqueous solution. The concept of prodrug based nanocarrier drug delivery system has gained a significant attention decade ago and is believed to propose pioneering clinical outcomes.^[^ ^12^ ^]^

Self‐assembling prodrugs are species of therapeutic agents having capability to associate into well‐organized supramolecular structures spontaneously in aqueous.^[^ ^13^ ^]^ Prodrug‐based drug delivery platform has become indispensable for disease treatments. It is generally thought that the oral administration of neuropeptides for treatment of brain disease is impossible because of the blood‐brain barrier, short blood half‐lives and poor oral absorption. This challenge can be tackled by the combination of nanotechnology and prodrug strategy. For instance, a palmitic prodrug of the drug leucine^5^‐enkephalin was formulated with chitosan (CS) into prodrug‐nanoparticle which showed a 67% increase of drug levels and drug's antinociceptive activity in brain,^[^ ^14^ ^]^ implying prodrug NPs enhance brain delivery by stabilizing the peptide in the plasma.

Self‐assembled drug delivery system (DDS)effectively combined the advantages of both prodrug strategy and nano DDS, such as improved drug loading efficiency, disease environment specific drug release, extended vascular circulation, and reinforced therapeutic efficiency. At first, most of prodrug is prone to self‐assemble into NPs by themself or with few supplementary materials, resulting in an ultrahigh drug loading without any carrier. Secondly, the self‐assembling prodrug nanomedicines (ProDNMs) show excellent biocompatibility and safety due to neglected surfactant addition. Thirdly, the simplicity of preparation, good stability, and optimized pharmaceutical properties provide unexpected convenience for further clinical applications. Finally, certain linkers not only help prodrugs assembly into nanostructures but also represent key structures dependent on the diseased cellular microenvironment. Convened above‐mentioned bullets together, ProDNMs enriched our arsenal to address unmet challenges from therapeutic agent's development domain. Typical self‐assembly prodrug consist of parental drug, linker and auxiliary segments.^[^ ^13^ ^]^ Among them, at least two components need to be included. The active drug is of course cornerstone, the linker and the auxiliary moiety are optional but of important parts. The controlled release of active pharmaceutical ingredient (API) is heavily depended on the feature of the linker; the self‐assembly of the conjugate is mainly determined by the property of the auxiliary moiety to achieve such as hydrophobic‐hydrophilic balanced prodrug, or interrupt the crystallization of pure API; In some case, the linker and the promoiety altogether affect the behavior of the conjugate; it is also hard to tell what the specific role of additional components toward API when only two parts appeared.

ProDNMs are particularly advantageous for cancerous treatments as they are often sufficiently small to internalize the cancer tissues but necessarily large to stay at the disease sites for therapy for relatively longer time, hence successfully fulfill so‐called the enhanced permeation and retention (EPR) effect. Rational combination of prodrug approaches with nanotech can synergistically improve the efficacy of cancer chemotherapy and lower/avoid the serious side effects of antitumor drugs.

Outnumbered side‐effects of anticancer drugs originate from their off‐target aftermath, meaning the chemical entities indiscriminately poisoned all cells regardless diseased or not.

Prodrugs are a proven route to limit off‐target exposure in tissues, in particular cytotoxic anticancer agents. Protection of the primary amine which is of importance for action of a drug, can be used to shield drug activity until the activity of drug is restored by spatiotemporally responsive deprotection. Nanoparticulation can achieve the same goals, and the brainstorm to marry prodrug and nanoformulation for controlling exposure in‐target versus off‐target in principle is striking. Drug delivery systems based nano structures spatiotemporal control strategy of in general has been inclusively discussed,^[^ ^15^ ^]^ therefore are not our priority hereof. Self‐assembling nanoprodrug for synergistic tumor targeted drug delivery is covered but not in depth discussed since it have been reviewed.^[^ ^16^ ^]^

**Scheme 1:**
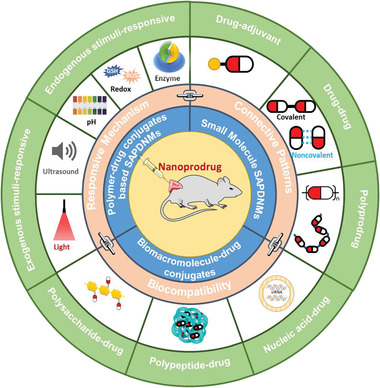
Schematic of the different forms of prodrug conjugates, respectively, and their potential as building blocks to fabricate nanoprodurg as newly emerging cancer therapy formulations under different endogenous/exogenous stimuli, drug connective patterns and biocompatibility.

We however started this contribution as summarized in **Scheme** **1** by discussing small molecule ProDNMs with defined structure, including drug‐adjuvant, drug–drug conjugate polyprodrug ProDNMs. Then detailed design strategies and applications of synthetic polymer‐drug conjugates based ProDNMs, exemplified by endogenous, exogenous cellular stimuli and multi‐stimuli responsive ones. Followed with the construction of biomacromolecule‐drug conjugates based ProDNMs. Lastly, we comprehensively envisioned the unmet challenges in this field, future directions, and the underdeveloped potential of nanoprodrug for cancer treatments. Expectantly, this work will spark further investigations of nanoprodrug in their clinical applications.

## 2 Small Molecule ProDNMs

From self‐assembling view, ProDNMs offer more tunability as any structurally‐driving intermolecular interactions introduced will perform an influence, enabling significant morphology control. Comparing to macromolecular peers, the key feature of small molecules self‐assembling prodrug are their precisely defined structure and homogeneous component since synthesized prodrug routinely experienced purification process. Moreover, the theoretical drug loading of ProDNMs can be as high as 100% at the circumstance of no any extraneous material being included, which therefore avoid related side effects and is more cost‐efficient because pharmaceutical excipients are supposed to be removed after their mission accomplished. However, complicated possibility of synthesis procedure in particular associated work‐up to fish out the pure prodrug and concerns of nanostructure stability during circulation should be also remembered.

Although small molecule ProDNMs can be roughly categorized into drug‐conjugates and drug amphiphiles, many structural similarities and overlap among them. The distinct difference is in their assembly and aggregation characteristics. In detail, drug conjugates require remarkable change in conditions and molecular structures to initiate assembly/aggregation, exemplified by swift mixing of drug conjugate in organic solvent which is water miscible into aqueous solution, or solvent replacement. Drug conjugate without the proper nanoprecipitation may fail to spontaneously assemble in water due to the unbalanced amphiphilicity, so require the external inducer for self‐assembling. However, optimized drug amphiphile favorably assemble into well‐defined supramolecular nanostructure with controlled size and shape when it is dispersed in aqueous solution.

### 2.1 Drug‐Adjuvant Conjugate

#### 2.1.1 Prodrug Nanoparticulation through Coupling with the Adjuvant

The concept of drug‐adjuvant prodrug has gained considerable attentions in recent years, and it usually obtained by a covalent coupling of the drug to biocompatible adjuvant moieties. The recipient substances have no direct therapeutic efficacy, but facilitate the formation prodrug NPs or assign other useful properties. Unsaturated fatty acids (UFAs), for instance, are biocompatible and tumor‐targeting, therefore were extensively considered for chemotherapy agent‐UFA (CA‐UFA) prodrugs design in cancer therapy. CA‐UFA prodrug‐based nanoparticulate drug delivery systems (DDS),^[^ ^17^ ^]^ which incorporate the merits of nano‐DDS and CA‐UFA prodrugs, have become a vivid field for the efficient delivery of chemotherapeutics. The advances of this strategy have been reviewed by Sun et al.,^[^ ^18^ ^]^ therefore is not covered herein.

Paclitaxel (PTX) is a commonly used chemodrug for diversity of tumors, exemplified by breast, ovarian, lung and pancreatic cancer, attributed to its role to block assembly‐disassembly dynamics of cellar microtubule polymerization. However, its poor solubility in water severely hampered its clinical application. So far, variety of attempts have been made to improve its solubility in aqueous.^[^ ^19^ ^]^ Intuitively, attaching the water solubilizing group to PTX might be a reasonable strategy to alter its physicochemical properties. Amino, carboxyl, hydroxyl, amino acids moieties hence were linked to PTX, achieved 6–140 folds increased water solubility and one prodrug exhibited better in vitro anticancer activity and remarkable superior bioavailability in mice comparing to those of PTX itself,^[^ ^19^ ^]^ but did not further developed into clinically valuable medicine. PTX was at first dissolved with using 50% Cremophor EL (CrEL) and 50% dehydrated ethanol to give commercial medicine Taxol. However, the severe side effects of Taxol are mainly related with CrEL. Similarly, Docetaxel formulation's side effects have been observed due to the utilization of ethanol and polysorbate 80. Encouraged by the wide anticancer spectra of PTX and its unique mechanism to inhibit cell growth, along with structural amendment, more and more attempts tried to combine medicinal chemistry and pharmaceutics approach to exploit the druggability of PTX in depth.

**Figure 1:**
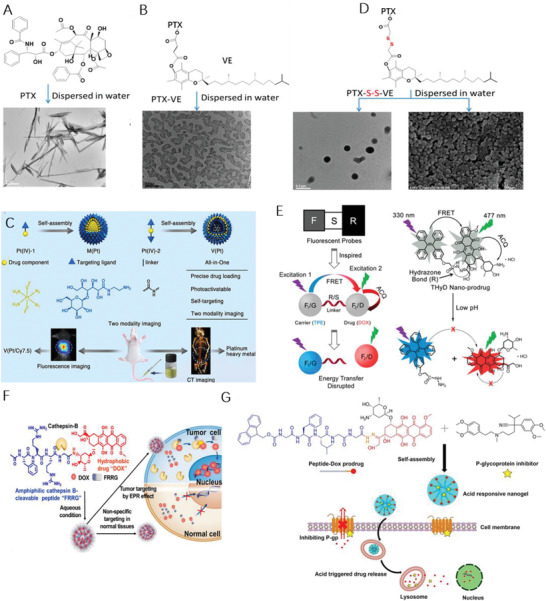
Molecular structures of A) PTX, B) PTX‐VE, and D) PTX‐S‐S‐VE and individual SEM/TEM images after dispersion in water. Reproduced with permission.^[^ ^24^ ^]^ Copyright 2014, American Chemical Society. C) Self‐targeting all‐in‐one platinum (IV) amphiphiles for multimodal theranostic precise nanomedcine. Reproduced with permission.^[^ ^21^ ^]^ Copyright 2018, American Chemical Society. E) Schematic illustration of the design of the probe‐inspired nanoprodrug. Reproduced with permission.^[^ ^26^ ^]^ Copyright 2015, American Chemical Society. F) Schematic illustration of the action of FRRG‐DOX NPs as new carrier‐free NPs for cancer targeting treatment. Reproduced with permission.^[^ ^27^ ^]^ Copyright 2019, Elsevier. G) Illustration of the co‐assembly of peptide‐DOX and P‐gp inhibitor into an acid‐responsive nanogel for controllable drug release to overcome cancer drug resistance. Reproduced with permission.^[^ ^31^ ^]^ Copyright 2017, Wiley‐VCH.

It is observed that hydrophobic molecules themselves hardly form stable NPs, represented by PTX alone assemble into large needles during self‐assembly (**Figure** **1**A). When PTX conjugated with vitamin E (VE) into a prodrug through succinate bond, sizeable aggregation upon aqueous exposure took place (Figure 1B), attributing to the increased hydrophobicity of the conjugate product. Phosphorylation is a widely used method to improve the water solubility of hydrophobic drugs. Hu et al. prepared a targeted lipid/calcium/phosphate NP formulation consisting of quercetin phosphate; the bioassay confirmed that quercetin phosphate alone significantly remodeled the tumor microenvironment and increased the penetration of NPs into the tumor matrix.^[^ ^20^ ^]^ The conjugated hydrophilic adjuvant to hydrophobic drug not only favored the self‐assembly, but navigated the assemble to diseased tissues. Depending upon the ratio of Pt/lactose moiety ratio, lactose containing platinum(IV) (Pt(IV)) amphiphiles self‐assemble into micelle or vesicle (Figure 1C), actualized precise drug loading, photoactivation, self‐targeting and two modality imaging all‐in‐one unit.^[^ ^21^ ^]^ The same strategy was utilized to increase the targeting and passive accumulation of anticancer drug doxorubicin (DOX)^[^ ^22^ ^]^ and camptothecin (CPT)^[^ ^23^ ^]^ profile in the tumor site. Besides, the insertion of single disulfide‐bond between the drugs keeps the hydrophobicity of the prodrug, but changes its behaviors in aqueous suspension as such the prodrug self‐assembled into NPs without additional excipients (Figure 1D),^[^ ^24^ ^]^ implying the importance of prodrug linker to concomitant self‐assemble profile in aqueous. Vitamin E was also covalently conjugated with DOX and low molecular weight PEG individually then co‐precipitated into 250 nm nanostructures with sustained in vitro release up to 6 month and higher in vivo antitumor regrowth delay when compared with free DOX.^[^ ^25^ ^]^ It is desirable to tracking the drug delivery kinetics in living cells, hence a self‐indicating nanoprodrug with two fluorescent moieties, connected by pH‐responsive linker was constructed^[26]^ to accurately followed the drug‐releasing site, the releasing time and the destinations of the drugs at subcellular level (Figure 1E).

Although the adjuvant has no direct therapeutical impacts, play critical role in other aspects of nanomedicines, such as targeting and morphology control. Shim et al. conjugated DOX with peptide Phe‐Arg‐Arg‐Gly into prodrug that formed a stable NP with average diameter of 213 nm, enabled tumor‐targeting (Figure 1F) and enhanced therapeutic efficiency in animal mode.^[^ ^27^ ^]^ Impressively, Santos et al. presented a tripodal boronate complexes featuring reversible covalent bonds to assemble anticancer drug bortezomib (BTZ), oligomer, and folate targeting units for cancer‐cell‐targeting drug conjugates.^[^ ^28^ ^]^ The morphology of adjuvant‐drug conjugate nanodrug is controllable by adjusting the structure of adjuvant. A small library of Irinotecan(Ir)‐derived prodrugs was constructed through esterification with fatty‐acid moieties which tuned the polarity of Ir and induced self‐assembly of Ir prodrug into NPs with different morphologies; they all accumulated in cancer tissues at higher levels and were much more cytotoxic than free drugs, in particular Ir‐lauric acid prodrug was most potent one.^[^ ^29^ ^]^ The nanoprodrug carrier system can also be constructed by blending one drug‐adjuvant with other adjuvants, for example, prodrug conjugate of anticancer drug gemcitabine (GEM) with protected glycine was noncovalently sandwiched by a pair of oppositely charged amyloid‐inspired peptides into nanofiber.^[^ ^30^ ^]^ One important factor attributing to multidrug resistance is overexpression of P‐glycoprotein (P‐gp) which lead to reduced intracellular drug accumulation. Effective block the P‐gp efflux to accumulate the therapeutics in cancer cells is a promising approach. Lyu et al. developed a peptide‐assembled nanogels (Figure 1G), which synergistically combined the acid‐activatable antitumor prodrug DOX with the P‐gp blocker verapamil to reverse multi‐drug resistant caner.^[^ ^31^ ^]^

#### 2.1.2 On‐Demand Release of Drug from Drug‐Adjuvant Conjugate ProDNMs

Apart from the feasibility of prodrug nanoparticulation through coupling with the adjuvant, the on‐demand release of parent drug from as‐prepared prodrug NPs is also crucial from clinical perspective. The PTX and docosahexaenoic acid prodrug conjugate has undergone phase 3 clinical trials,^[^ ^32^ ^]^ but far behind expectation because of the staggering ester bond hydrolysis related extremely slow PTX release.^[^ ^33^ ^]^ Given the observation of the intracellular glutathione (GSH) level in most cancer cells was abnormally higher than disease‐free tissue,^[^ ^34^ ^]^ The redox‐liable NPs drug delivery systems has been extensively investigated. The disulfide bond bridged PTX and oleic acid (OA) prodrug‐based self‐assembled into nanocomposites (PTX‐S‐S‐OA) which fulfilled quick and adjustable release of PTX in redox environment.^[^ ^35^ ^]^ Disulfide bond linked CPT and palmitic acid prodrug conjugates can formed NPs and achieved high oral absorption and reduced intestinal side effect for cancer treatment.^[^ ^36^ ^]^ To improve the clinical potential of CPT based nanoformulaiton in tumor therapy, phosphorylcholine was introduced into CPT prodrug conjugates.^[^ ^37^, ^38^, ^39^ ^]^

Tumor cells are heterogeneous with respect to redox profile, meaning abnormal GSH and ROS may coexist in different regions of tumor, present among different tumors or different levels of GSH/ROS were found at different tumor stage.^[^ ^40^ ^]^ Nevertheless, most stimulus‐sensitive nano‐DDS responded to overproduced either GSH or ROS fail to fully deteriorate therapeutic potential. Having known the redox dual‐responsiveness^[^ ^41^ ^]^ and mechanism^[^ ^42^ ^]^ of dithioether, Luo et al. postulated that single thioether was superior to dithioether as a redox dual‐sensitive bridge. Two PTX oleic acid conjugate prodrugs (PTX‐S‐OA and PTX‐2S‐OA) nanocomposites via single thioether and dithioether linker hence were individually synthesized.^[^ ^43^ ^]^ Attributing to the effectiveness of thioether oxidized into sulfoxide or sulfone, about 46% of PTX released from PTX‐2S‐OA, while more than 90% of PTX‐S‐OA was hydrolyzed within 6 h in the presence of 10 × 10^−3^ m H_2_O_2_ over 12 h. Consistently, the release rate of PTX from PTX‐S‐OA was almost double comparing to the PTX‐2S‐OA nanocomposites when 10 × 10^−3^ m dithiothreitol (DTT) was charged.^[^ ^43^ ^]^ It was also reported that thioether bond and disulfide bond in prodrugs were better‐performed than ester bond‐linkage against anticancer therapy, for instance DTX‐maleimide conjugate prodrug with disulfide or thioether bond linkages were 30‐folds more selective comparing with ester bond in tumor tissue than in liver.^[^ ^44^ ^]^ In addition, these prodrugs can be further masked through the Michael addition reaction between the albumin and the prodrugs, consequently improved the bioavailability of drug 21‐fold and drug accumulation in tumor 8‐fold comparing to free one.^[^ ^45^ ^]^

**Figure 2:**
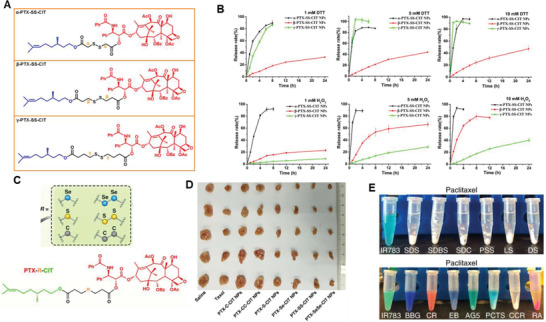
A) Chemical Structures of the Disulfide‐Bond‐Bridged PTX‐CIT Prodrugs: *α*‐PTX‐S‐S‐CIT, *β*‐PTX‐S‐S‐CIT, and *γ*‐PTX‐SS‐CIT; B) In vitro redox dual‐responsive drug release of prodrug nanoassemblies in the presence of various concentrations of DTT or H_2_O_2_ (*n* = 3). Reproduced with permission.^[^ ^46^ ^]^ Copyright 2018, American Chemical Society. C) Sulfur/selenium/carbon bond‐bridged PTX‐CIT prodrug. D) Images of tumors after in vivo antitumor treatment using different types of prodrug nanoassemblies. Adapted with permission.^[^ ^48^ ^]^ Copyright 2019, Nature Publishing Group. E) Result of attempted water dispersion of paclitaxel with a panel of excipients. Reproduced with permission.^[^ ^54^ ^]^ Copyright 2018, Nature Publishing Group.

Further on, it might be reasonable to question that is disulfide bond also redox dual‐sensitive in cancer cell microenvironment? Does the position of disulfide bonds among linkage impact its redox behavior? To address these issues, Sun et al. synthesized three PTX‐citronellol conjugates linked via different lengths of carbon chain with disulfide‐bond inserted at different position (**Figure** **2**A), these prodrugs self‐assembled into NPs which showed redox dual‐responsive drug release. Moreover, the disulfide bond's position in the carbon chain significantly affected the redox dual responsivity, with the slowest PTX release from *β*‐PTX‐S‐S‐CIT NPs in the presence of DTTand *γ*‐PTX‐S‐S‐CIT NPs when H_2_O_2_ appeared (Figure 2B) due to different degradation mechanism, thereby impacted the biodistribution, cytotoxicity, pharmacokinetics, drug release profile and in vivo antitumor efficacy of prodrug NPs.^[^ ^46^ ^]^ Encouraged by the redox‐responsiveness of sulfur containing bonds, selenium (Se), belonging to the same sixth family as sulfur in the elements periodic table and being essential trace element in the antitumor treatments,^[^ ^47^ ^]^ was also of increasing interest for cancer treatment application. It was hypothesized that the insertion of a single disulfide bond into hydrophobic molecules balanced the competition between intermolecular forces since disulfide bond prefer dihedral angles of near 90°, disrupted crystal growth but stabilized nanomedicines formation of prodrug.^[^ ^24^ ^]^ Continuously, six PTX‐citronellol conjugate analogs (Figure 2C) with thioether bond, disulfide bond, selenoether bond, diselenide bond, carbon bond or carbon–carbon bond containing linkage were synthesized;^[48]^ As expected, these prodrugs all self‐assembled into NPs, but the efficiency of individual nanoassembly, stability pharmacokinetics, even drug release and even cytotoxicity dependent on sulfur/selenium/carbon bond angles/dihedral angles. For instance, PTX‐SeSe‐CIT NPs exhibited the most potent tumor‐inhibiting activity hence significantly reduced tumor volume (Figure 2D).^[^ ^48^ ^]^

The squalenoylation, an antitumor strategy with enhanced activity,^[^ ^49^ ^]^ was employed to improve pharmacokinetical profiles of both hydrophilic and hydrophobic therapeutic agents.^[^ ^50^ ^]^ The quick metabolization and rapid clearance heavily compromised neuropharmacological activity of adenosine upon systemic administration. Hence adenosine was conjugated with squalene and the assembled into NPs which prolonged the circulation of the nucleoside and protected the neuro of spinal cord injured rat.^[^ ^51^ ^]^ In similar, when squalene coupled to hydrophobic active compounds, including PTX, podophyllotoxin, camptothecin (CPT) and epothilone A through both disulfide or all‐hydrocarbon bond to form NPs spontaneously in water, and release the parent drug in vitro, but the size, stability and in vitro activity differed.^[^ ^52^ ^]^ The monoisoprenoyl was conjugate to PTX to give prodrug, which was further self‐assembled into NPs through the surfactant squalene derivative.^[^ ^53^ ^]^

The nano‐sized prodrugs often need to experience covalent synthetic schemes to stabilize the NPs formulation of parent drug, it was also shown that nanomedicines can be obtained through non‐covalent interactions between target drug and supplementary/complementary substances. For example, Yosi et al. reported that indocyanine IR783 enable the formation of stable PTX prodrug NPs with 84% of drug loading by simple physical mixing. Besides, eight out of sixteen resulted in monodispersed NPs suspension with different colors (Figure 2E), implying tendency to form NPs is structure dependent. Consequently, the quantitative structure‐property relationship analysis was performed to identify main molecular descriptors of drugs which correlated with the successful formation of prodrug NPs. The robustness of this correlation was also validated as such that about 98% consistency between prediction and tested NPs formation was predicted,^[^ ^54^ ^]^ these findings suggest the rational design of nanomedicines based on quantitative models is applicable and could extend to computational pharmaceutics. However, to what extent the computational prediction can be applied remains unknown, attributing to limited data collection, overlooked molecular descriptors, ignored principal components analysis, which all determine the accuracy of prediction. In addition, the above‐mentioned exploration is based on the non‐covalent interactions between prodrug components while covalent prodrugs conjugates are more popular to date. Yet none systemic computational investigation for such prodrug nanomedicines although some molecular dynamics has been performed.^[^ ^55^ ^]^

Cancer stem cells (CSCs) show resistance to treatment and cause tumor recurrence. Misra et al. conjugated CSCs inhibitor nifuroxazide with phosphocholine then added to polyethylene glycolcetyl ether micellar assembly to form Pro‐nifuroxazide NPs with sub 20 nm size; achieved 2.4‐folds more effective for cancer cells inhibition compared to parent inhibitor, attributing to ≈240 folds improvement in the local concentration of drugs when formulated into prodrug NPs.^[^ ^56^ ^]^ To formulate active pharmaceutical ingredient into nanomedicine, excessive excipients is often used, therefore lead to low drug loading. To increase the anticancer drug proportion in specific formulation, Shen et al. fabricated nanocarriers that CPT molecule(s) were conjugated to a short ethylene glycol oligomer chain, forming stable liposome‐like vehicle with a drug loading of 58 wt%; this nanocapsules also enable the co‐encapsulation of water‐soluble DOX in the hydrophilic center of vehicle for combination therapy.^[^ ^57^ ^]^

**Figure 3:**
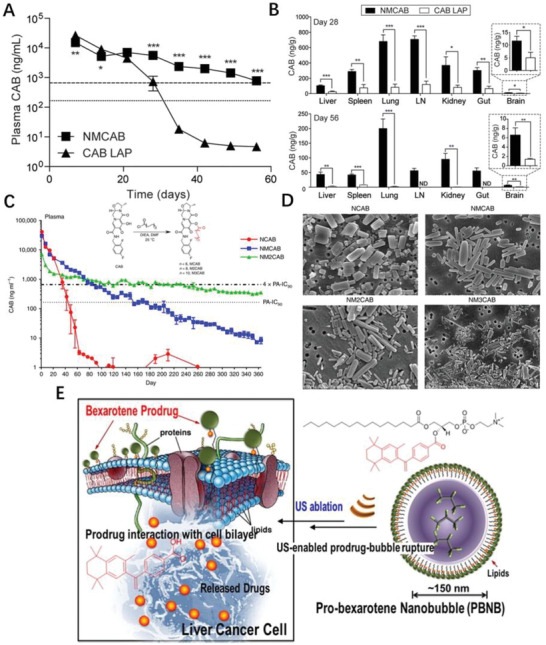
A) Plasmas were isolated at each blood collection, and then analyzed for CAB concentrations by UPLC/MS/MS. Horizontal dotted and dashed lines represent 1  ×  and 4  ×  PA‐IC90, respectively. B) Tissues were collected at each sacrifice day and then analyzed for CAB concentrations by UPLC/MS/MS. LN: lymph node. Reproduced with permission.^[^ ^58^ ^]^ Copyright 2018, Elsevier. C) CAB was chemically modified with 14‐, 18‐ and 22‐carbon fatty acid chains to develop MCAB, M2CAB, and M3CAB, respectively. D) Morphological assessment of NCAB, NMCAB, NM2CAB, and NM3CAB by SEM. Reproduced with permission.^[^ ^59^ ^]^ Copyright 2020, Nature Publishing Group. E) Layered arrangement of bexarotene prodrug after self‐assembling into a gas‐filled bubble followed by its rupture upon US exposure and interacts favorably with the cancer cell membrane to get inserted and eventually be cleaved enzymatically, releasing the active pharmaceutical ingredient. Reproduced with permission.^[^ ^60^ ^]^ Copyright 2015, American Chemical Society.

Prodrug(s)/prodrug formulation will inevitably hamper the liberation of parent drug either the active components were physical trapped in or chemical conjugated with carriers. Such decelerated release profile originated from nanoformulation is particularly useful for suppression of viral replication. To improve the cabotegravir (CAB) delivery profile, Zhou et al. synthesized a myristoylated CAB prodrug (MCAB), subsequently formulated into NPs; the injection of the NPs maintained the CAB plasma drug concentrations above 4 X protein‐adjusted 90% inhibitory concentration (PA‐IC_90_) for up to 60 days (**Figure** **3**A);^[58]^ at the equal injection concentration of CAB for animal study, up to 95‐fold increases in CAB concentrations were detected in liver, lungs, spleen and lymph nodes in NMCAB treated animals (Figure 3B). Although current long‐acting CAB significantly decease frequency of administration from days to months, broader application is hurdled by health‐care carelessness, injection site responses and dosing volumes. To tackle this challenge, fatty acid‐CAB prodrugs with 14, 18, and 22 added carbon chains were developed (Figure 3C) and manufactured into uniform rod‐shaped NPs (Figure 3D); amongthem, 18‐carbon products generated CAB plasma concentrations above the PA‐IC_90_ inhibitory concentration of 166 ng mL^−1^ for up to one year,^[^ ^59^ ^]^ which dramatically prolonged apparent half‐life of small chemotherapeutics, could be consider as a potential vaccine mimetic.

A brand‐new drug for disease‐treating through a classical drug discovery streamline is time‐consuming and cost‐ineffective. One approach is drug repurpose, intended to find alternative uses for approved drug or a drug that is made by another innovator. The combination of drug repurposing and prodrug strategy has also led to novel nanomedicine. For example, bexarotene is used for cutaneous manifestations of T‐cell lymphomas, while re‐engineered bexarotene prodrug made of hydrophobic acyl chain, hydrophilic lipid head and bexarotene self‐assembled into pro‐bexarotene nanobubble (Figure 3E) which favorably approached and inserted into the cancer cell membrane and eventually be cleaved enzymatically, releasing the active bexarotene.^[^ ^60^ ^]^

#### 2.1.3 Theranostic Drug‐Adjuvant Conjugate ProDNMs

Apart from therapeutic prodrug nanomedicines, the theranostic prodrug nanoassembles are also desired for improved diagnostic precision and therapeutic efficacy.^[^ ^61^ ^]^ Near‐infrared (NIR) theranostic prodrug for cancer therapy and imaging was developed by Kong et al.^[^ ^62^ ^]^ Chen et al. developed prodrug agents which covalently combined fluorescent reporters cyanine 5.5 with chemotherapeutic agents cyclopeptide RA‐V through disulfide bond and assembled into NPs; linked together, the fluorescence of Cy5.5 was quenched therefore in “turned‐off” state while “turned on” when the disulfide linker was cleaved by high GSH disease areas hence monitoring drug release and chemotherapeutic efficacy in situ.^[^ ^63^ ^]^ Beside the facilitating NPs formation and diagnostic functions, the adjuvant part of prodrug can also improve the pharmacokinetics and biodistribution of parent drug in tumor. CPT‐albumin binding Evans blue prodrug self‐assembled into 80 nm of NPs that quickly altered to 7 nm albumin/prodrug nanocomplexes after being administratored, the nanocomplexes were efficient untaken into HCT116 colon cancer cells, exhibited 130‐fold greater long blood circulation than CPT and 30‐fold tumor accumulation of CPT in animal mode.^[^ ^64^ ^]^

**Figure 4:**
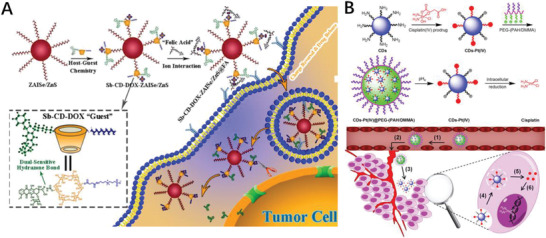
A) Overall Synthetic Scheme for Sb‐cyclodextrin(CD)‐DOX‐ZAISe/ZnS@FA NPD by “Host–Guest” Assembly and Electrostatic Interaction Strategy. Reproduced with permission.^[^ ^65^ ^]^ Copyright 2018, American Chemical Society. B) Schematic illustration for the preparation of charge‐convertible CDs‐based drug nanocarrier CDs‐Pt(IV)@PEG‐(PAH/DMMA) and the drug delivery process of CDs‐Pt(IV)@PEG‐(PAH/DMMA) Reproduced with permission.^[^ ^66^ ^]^ Copyright 2016, American Chemical Society.

Nanoprodrug was also incorporated with other imaging/therapeutical agents to fulfill theranostic functions. Taking the advantage of quantum dots (QDs) in tumor imaging and drug tracking properties, Deng et al. constructed a ZnAgInSe/ZnS QD‐based theranostic prodrug via host‐guest interaction which enable precise intermolecular self‐assembly; beside the high‐resolution imaging for malignant tumors, folic acid coated QDs nanodrug internalized the tumor cell by undergo charge reversal (**Figure** **4**A), effectively inhibited the tumor growth up to 50%, further confirmed by histological observation.^[^ ^65^ ^]^ Positive charged carbon dots were decorated by negative charged cisplatin(IV) prodrug through ionic interactions and further masked with anionic polymer as nanocarrier which underwent charge conversion and drug release (Figure 4B) to respond acidic tumor extracellular microenvironment.^[^ ^66^ ^]^

The numerous self‐assembly of drug‐adjuvant conjugates into NPs has been described, but the detailed assembling mechanism is poorly understood. Taking PTX born ones as example, the PTX itself precipitated immediately but some of its derivatives, including hydrophobic moieties attached ones form monodispersed particles when the same nanoprecipitation process was applied. Obviously, the hydrophobic fragments conjugated to PTX play critical roles in the self‐assembly. What in common among these external hydrophobic moieties is they all bear double bond or phenyl group, which may indicate these bulky group facilitate the intermolecular stacking interaction with the planar structures of PTX. Experiments also showed the XlogP, Hansen solubility parameters, together with amorphous state of PTX‐small molecule modified conjugates were critical for self‐assembling into NPs.^[^ ^67^ ^]^

### 2.2 Drug–Drug Conjugate ProDNMs

The key mission of pharmaceutics is to effectively deliver the medications to the diseased tissue according to spatiotemporal demanding. Since any recipient will anyway be considered as xenobiotics therefore possibly cause unwanted immunological exclusion. In addition, the adding of recipient is not economically efficient. Therefore, prodrug nanomedicines without drug carriers have attracted many attentions as excipient‐free strategy will intuitively offer the maximum drug loading. Comparing to physical encapsulation, drug–drug conjugation methods might be preferred because conjugating drug one another can produce more stable prodrugs with only active ingredients, which minimize the possible toxicity induced by other exogenous materials^[^ ^68^ ^]^ and side effects due to immature drug release.

Apparently, not all two/several drugs are capable of forming a suitable prodrug, leaving a ProDNMs alone. Therefore some of prerequisite should be considered for successful prodrug synthesis and following nanofabrication: 1) the possibility of covalent conjugation, means the drugs need to possess reactable group, such as alcohol, carboxylic acid and amine to enable condensation reaction take place; 2) synergistic outcome of the monomeric drug candidates is always pursued; 3) a balance of hydrophobicity and hydrophilicity is desired as a bias will deteriorate the NP formation; 4) the essential dissociation of the prodrug in biological microenvironment; 5) the possibility to follow the trajectory of the processed prodrug NPs. Based on these principles, considerable drug–drug conjugates have been designed abiding by the rationale that integrated drugs with different mechanism of action and reactable functional moieties to improve individual therapeutic efficacy or/and avoid drug resistance.

#### 2.2.1 Chemical Reaction Obtained Drug–Drug Conjugate ProDNMs

Noncovalent coencapsulation of multiple small chemodrugs into a single NP platform is widely investigated, but may not achieve promising results because of drug loading competition inside the NPs and insufficient stability of the whole nanosystem. Therefore, one move attention to the covalent marriage of small drug molecules as prodrug then produce therapeutical NPs.

**Figure 5:**
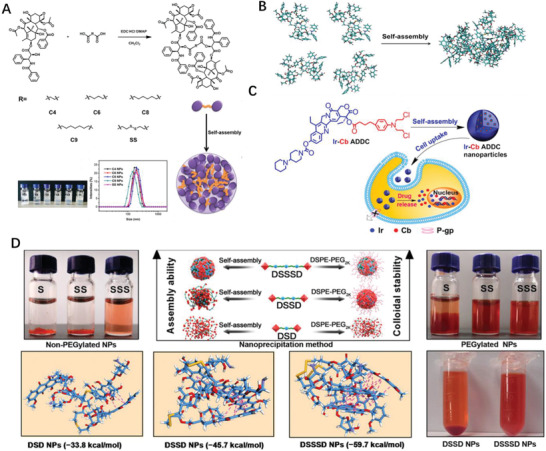
A) Synthesis of PTX_2_ and Self‐assembly of PTX_2_ in aqueous solution. Reproduced with permission.^[^ ^69^ ^]^ Copyright 2017, Elsevier. B) MD simulations of tetrameric PTX‐S‐S‐PTX in water. Reproduced with permission.^[^ ^70^ ^]^ Copyright 2016, American Chemical Society. C) Schematic diagram of amphiphilic drug–drug conjugate from fabrication, self‐assembly to self‐delivery. Reproduced with permission.^[^ ^73^ ^]^ Copyright 2014, American Chemical Society. D) Preparation and characterization of DOX homodimeric prodrug nanoassemblies. Reproduced with permission.^[^ ^80^ ^]^ Copyright 2020, AAAS.

Although PTX itself failed to form nanosized particles, dimeric PTX behave differently. Coupling two PTX molecules with dicarboxylic acids arose a series of PTX dimers which formed stable NPs (**Figure** **5**A) in aqueous solution, and the maximum solubility of 1000 µg mL^−1^ was achieved by using PTX_2_ NPs formulations, a 2500‐folds increasing compared to that of free PTX; more importantly, these dimeric PTX NPs exhibited comparable cytotoxicity with commercial drug Taxol.^[^ ^69^ ^]^ Han et al. reported the PTX‐S‐S‐PTX conjugate self‐assembled into uniform NPs which also encapsulated lipophilic fluorescent dye as core to facilitate the synergetic chemo‐thermal therapy; further molecular dynamics (MD) simulations shown four PTX‐S‐S‐PTX molecules gathered to form a cluster of tetramers as such the phenyl rings of PTX were curved inside the cluster and the driving forces for the self‐assembly were noncovalent hydrophobic interactions and *π*−*π* stacking between dimeric PTX (Figure 5B).^[^ ^70^ ^]^ PTX‐S‐S‐PTX prodrug dimer was also trapped into copolymeric biomaterial NPs to improve the outcome of chemotherapy.^[^ ^71^ ^]^ By coupling two curcumin (CUR) molecules with a thioether linker into CUR‐S‐CUR then self‐assemble into spheric NPs in aqueous solution, which enable enhanced intracellular CUR delivery and GSH‐responsive delivery.^[^ ^72^ ^]^

Except the homodimeric drug conjugate, two different types of drugs can also “marry” to form a prodrug unit. The hydrophilic anticancer drug irinotecan (Ir) bearing hydroxyl moiety bearing and the hydrophobic drug chlorambucil (Cb) with carboxylate containing was directly conjugated through esterification as amphiphilic prodrug, then self‐assembled into monodisperse NPs which enter tumor cells through endocytosis; the cleavage of the ester bond by esterase triggered the release of Ir and Cb in tumor cells (Figure 5C).^[^ ^73^ ^]^ Referring to the approach, we also fabricated anticancer drug PTX and DOX prodrug conjugates into NPs which enable the selective killing of DOX resistant cell line MDA‐MB‐231/ADR;^[^ ^74^ ^]^ the similar formulation for lung cancer therapy.^[^ ^75^ ^]^ The combination of hydrophilic drug methotrexate (MTX) with the hydrophobic anticancer drugs camptothecin (CPT) and DOX through disulfide bond and hydrazone bond were also reported.^[^ ^76^ ^]^ By doping with a lipophilic NIR cyanine dye DiR, the MTX‐CPT NPs was effectively probed for in vivo NIRF/PA dual‐modal imaging.^[^ ^77^ ^]^ Attributing to its amphiphilic property, CPT‐S‐S‐Gemcitabine(GEM) prodrug could self‐assemble into Janus NPs in water and released more than 90% of native drug with 3 h and showed broader synergistic/concurrent anticancer proliferation in vitro bioassay.^[^ ^78^ ^]^ The similar design of CPT and cytarabine conjugate also realized synergistic chemotherapy effects.^[^ ^79^ ^]^

It is also shown the linker between drugs significantly affects the features of prodrug conjugate. For instance, compared with disulfide and thioether linkers, the trisulfide bond effectively promotes the self‐assembly ability of DOX homodimeric prodrugs (Figure 5D), thereby improving the colloidal stability and the fate of prodrug nanoassemblies; further MD simulation disclosed that the trisulfide bond had more sulfur‐containing bond angles, and were close to 90°,^[^ ^80^ ^]^ which were believed the optimal angle to constructed the most stable conformation, and kept agreement with the lowest calculated free energy.^[^ ^80^ ^]^ Amino acid valine linked PTX and MTX to offered prodrug conjugate then assembled into NPs.^[^ ^81^ ^]^

It is also found that the drug–drug conjugate may change the mechanism of action, exemplified by temozolomide (TMZ)‐Dox prodrug double intercalating with DNA although TMZ itself is a weak DNA binder and functioned as an induced intercalator.^[^ ^82^ ^]^

#### 2.2.2 Drug–Drug Nanoparticulation through Physical Blending/Mixing

**Figure 6:**
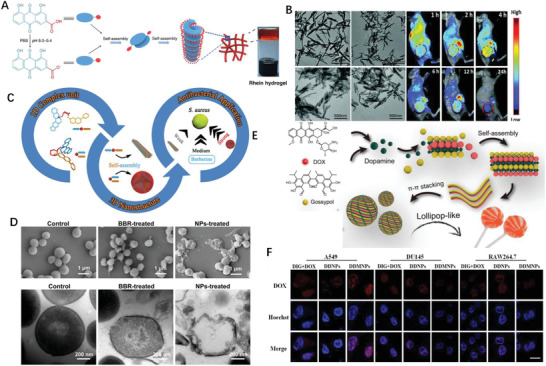
A) Self‐assembly process diagram of the Rh hydrogel. Reproduced with permission.^[^ ^83^ ^]^ Copyright 2019, Nature Publishing Group. B) TEM images of HCPT/Ce6 hybrid nanostructures with different ratios and whole body fluorescence images of 4T1 tumor‐bearing mice at different time points after being treated with HCPT/Ce6 NRs via intravenous injection. Reproduced with permission.^[^ ^84^ ^]^ Copyright 2017, Royal Society of Chemistry. C) Natural self‐assembling mode between berberine (BBR) and flavonoid glycosides (BA, WOG) and their modified antibacterial application. Reproduced with permission.^[^ ^86^ ^]^ Copyright 2019, American Chemical Society. D) Antibacterial mechanism: (top) Interaction between CA‐BBR NPs and MRSA. Inset shows HPLC‐HRMS/MS analysis of BBR in MRSA. (middle) FESEM images of MRSA treated without or with BBR and CA‐BBR NPs. (bottom) TEM images of MRSA treated without or with BBR and CA‐BBR NPs. Reproduced with permission.^[^ ^87^ ^]^ Copyright 2020, American Chemical Society. E) Schematic illustration of the design and prepared DOX‐dopamine‐gossypol NPs with ultralong blood circulation and enhanced tumor penetration for efficient synergistic chemotherapy. Reproduced with permission.^[^ ^89^ ^]^ Copyright 2019, Wiley‐VCH. F) Confocal fluorescence images of cancer‐derived NPs after 6 h co‐incubation with cells. Reproduced with permission.^[^ ^92^ ^]^ Copyright 2021, Elsevier.

Chemical modification of the small therapeutical regimens necessitate the development of excipient‐free NPs, which may also compromise the therapeutic outcome of the component drugs as the original features are changed anyway. Yet the covalent connection of drug–drug conjugates is not the precondition for NPs formation, drug pairs with complementary merits could form nanostructure non‐covalently. This strategy has been also applied for pure drug molecules nanofabrication and may achieve nearly 100% loading of the small molecular drugs avoiding the excipients use alike. In most simple case, the active components can self‐assemble into nanostructure by itself under suitable condition. For instance, Rhein (Rh) alone self‐assembly into hydrogels (**Figure** **6**A) consists of 3D NFs network and exhibited sustained drug release, easy cell internalization and significantly eased neuroinflammation without cytotoxicity in comparison with the equal amount of free‐drug.^[83]^ Notably, the driving forces of above‐mentioned self‐assembling process are hydrogen bonds, *π*–*π*, electrostatic and hydrophobic interactions, depending on molecular structure of individual components. The supramolecular self‐assembly could occur by physical mixing as long as the constituent is structurally complementary. Wen et al. directly assembled hydrophobic anticancer drug 10‐hydroxycamptothecin (HCPT) with a photosensitizer chlorin e6 (Ce6) to form carrier‐free nanorods (NRs); the as‐prepared NRs efficient internalized into and killed several kinds of cancer cell through chemophotodynamic dual therapy, almost completely inhibit the tumor growth in animal model (Figure 6B).^[^ ^84^ ^]^ Some anticancer drug itself, such as irinotecan hydrochloride is amphiphilic can therefore function as surfactants to co‐fabricate stable excipient‐free nanoplatform of water insoluble drugs for cancer therapy.^[^ ^85^ ^]^

The attempts to construct drug–drug nanocomposite based on dual components has been widely investigated, multiple components could also be added to form more advanced system.

Inspired by the compatibility of traditional Chinese medicine, Li et al. reported two self‐assembling patterns between berberine (BBR) and flavonoid glycosides baicalin (BA) and wogonoside (WOG) (Figure 6C); BBR‐BA assembled into spheritic NPs with hydrophilic moiety toward the outside and exhibited stronger affinity to bacteria, thereby remarkably improved bacteriostatic activity than BBR itself; whereas BBR‐WOG formed nanofibers (NFs) displayed a much weaker effect than BBR.^[^ ^86^ ^]^ BBR and cinnamic acid also directly formed into NPs who elucidated better inhibitory effect on multidrug‐resistant *S. aureus* (Figure 6D) and stronger biofilm removal ability.^[^ ^87^ ^]^ The above‐mentioned NPs’ self‐assembly was driven by ionic, hydrogen bonds and *π*–*π* stacking interactions which triggered the NPs’ formation of smallest units then layered 3D spatial configuration. Thanks to intermolecular interactions, integrating PEGylate indoleamine‐2,3‐dioxygenase inhibitor and photosensitizer (Indocyanine green) has led to a core–shell nanostructure with less 40 nm in size.^[^ ^88^ ^]^ Wang et al. prepared lollipop‐like dual‐drug‐loaded NPs consisted of gossypol, DOX and dopamine via *π*–*π* stacking, in which polydopamine filled into the space between the gossypol and DOX molecules, and formed into super long‐circulating (>192 h) particles (Figure 6E).^[^ ^89^ ^]^ What can learn from these cases is that the structural difference of self‐assembly nanomedicines related with different activity even the chemical components are identical or slightly differ, therefore we need to design favorable self‐assembly and avoid unfavorable one simultaneously.

DOX, a genotoxic anticancer drug, exerts anticancer effects by creating various DNA lesions on cancer cells. Regrettably, cancer cells could remove these DNA damages through their DNA damage repair network. Studies showed that inhibition of the DNA damage repair could sensitize cancer cells to DOX.^[^ ^90^ ^]^ Zhang et al. demonstrated that digoxin (DIG) could enhance the anticancer effect of DOX on lung cancer cells using the same mechanism,^[^ ^91^ ^]^ but with narrow therapeutic window (not higher than 2.8 ng mL^−1^); DOX causes toxicity in the liver and heart due to the lack of tumor targeting; additionally, carrier‐free NPs are easily aggregated or rejected by the defense system of body. To address these challenges, DIG and DOX were incorporated into nanomedicine via the reprecipitation procedure, then modified with A549 cell membranes to build cancer‐derived NPs which specifically internalized into A549 cells but not to other cells (Figure 6F).^[^ ^92^ ^]^

Due to the rapid plasma degradation, therapeutic efficacy of GEM is heavily compromised. Compounded by its hydrophilic nature, the efficient encapsulation into nanocarrier systems and sustained release are also challenge. Few trials of chemical‐physical interaction hybrid prodrug which integrate more anti‐cancer elements also showed impressive results. Co‐assembly between pemetrexed and cytosine‐containing diselenide into NPs which was decomposed when *γ*‐radiation applied, leading to the release of pemetrexed; meanwhile, diselenide was oxidized to seleninic acid which suppressed the expression of human leukocyte antigen E in cancer cells, thus fulfilled combined immuno‐, radio‐, and chemotherapies for cancer treatment.^[^ ^93^ ^]^ MTX is folic acid like chemodrug and could bind to folate receptors due to the structural similarity. Hence self‐targeting nanodrug consist of MTX and HCPT was developed and demonstrated the morphology, size of the nanodrug is tunable by changing the assembly time and drug‐to‐drug ratio.^[^ ^94^ ^]^ An indocyanine green (ICG)‐templated self‐assembly strategy first got PTX involved, then was generalized to screen a library of small molecular drugs including kinase inhibitors, receptor antagonist, ion channel blocker, antihyperlipidemic drug and immune regulator.^[^ ^95^ ^]^

### 2.3 Polyprodrug Nanomedicines

Variety of natural and artificial pharmaceutical excipients have been utilized to construct nanodrug delivery systems; among them the polymer is in dominant status. However, few polymeric drug carriers have biological activity even some of them bear side effects. Therefore, it would be beneficial if the polymer itself could provide therapeutic effects.

**Figure 7:**
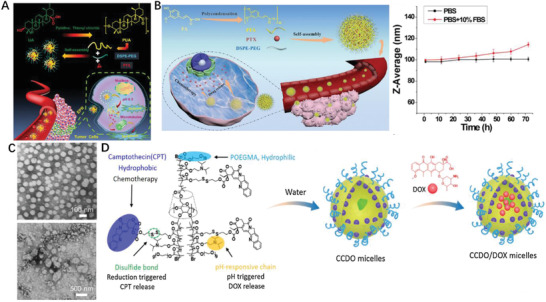
A) Illustration of major steps about PTX delivery by PUA‐NPs@PTX to colorectal cancer. Reproduced with permission.^[^ ^96^ ^]^ Copyright 2020, Wiley‐VCH. B) In vivo imaging of the PFA loaded PTX NPs biodistribution in CT26 tumor‐bearing mice. Reproduced with permission.^[^ ^98^ ^]^ Copyright 2019, Wiley‐VCH. C) Pharmacokinetics and biodistribution of HCPT‐based polyprodrug NPs. Reproduced with permission.^[^ ^99^ ^]^ Copyright 2020, Elsevier. D) Overlaid fluorescent images of the HepG2 tumor‐bearing nude mice at 24 h post‐injection of the NPs of P(HCPT/DTDE)‐b‐PEG‐COOH, RGD‐NPs and free GGRGD 30 min followed by RGD‐NPs (RGD+RGD‐NPs). Reproduced with permission.^[^ ^101^ ^]^ Copyright 2019, Elsevier.

From chemistry perspective, therapeutical reagents bearing polyfunctional groups could be self‐polymerized and assigned new utilities. Most botanical derivatives are bifunctional hence are ideal polymerizable monomers. Additionally, if those components are implied with health benefits, they would be will potential building block of therapeutics. Studies reported ursolic acid (UA) bearing hydroxyl and carboxylic acid moiety can inhibit the proliferation of various cancer cell types. UA was recently polycondensed as poly(ursolic acid) (PUA) and can self‐assembled into NPs in which PTX was trapped (**Figure** **7**A).^[^ ^96^ ^]^ In similar, ferulic acid, a hydroxycinnamic acid was chemically modified to form poly(ferulic acid) (PFA) whose DOX^[^ ^97^ ^]^ and PTX[98] loading formulation can also self‐assemble into NPs remained stable with 70 h, while the size varied between 100 and 120 nm over 3 days in 10% fetal bovine serum (Figure 7B). Those self‐assembled NPs effectively deliver chemodrugs, also provide additional therapeutic effects, therefore is regarded as a promising platform against cancer treatments. Not only can polyprodrugs themselves self‐delivered in nanocomposite form, but also function as drug delivery carrier. A NP drug delivery vehicle made of redox‐responsive HCPT polyprodrug inner core, amphiphilic lipid‐poly (ethylene glycol) outer shell, and lactobionic acid on the surface as targeting moieties was reported by Li et al.; the resulting NP with the size below 100 nm changed its morphology from spherical shape into amorphous aggregates in GSH depending manner (Figure 7C).^[99]^ Superfast redox‐responsive drug release (around 70% HCPT liberation within 2 h)^[^ ^100^ ^]^ and dramatic inhibition of tumor growth without side effects. Besides, *β*‐CD based polyprodrug, contained a hydrophilic polymeric chain and a hydrophobic part of CPT prodrug (Figure 7D), could form stable unimolecular micelles and enabled the encapsulation of DOX into the hydrophobic center.^[^ ^101^ ^]^ A guanidinium‐pendant Pt(IV) polyprodrug nano‐assemble released toxic Pt(II) species under the intracellular reducing conditions and significant increase the survival rate of treated mice.^[^ ^102^ ^]^ The above‐mentioned chemodrugs with multiple reactive functional moieties can be polymerized hence present unique advantages, for instance polyprodrug can serve as the carrier of another drug. Inspired by these results, one may think other drug monomers with bi/multifunctional group, such hydroxyl, carboxylic acid and amine are likely polymerizable. Therefore, it is worth taking other dual reactive function moieties reagents such as oleanolic acid (OA), and betulinic acid into consideration for polyprodrug synthesis and concurrent nanofabrication.

## 3 Polymer‐Drug Conjugates Based ProDNMs

The feature of the ProDNMs being they are made of small molecules alone and a well‐defined self‐delivery system, but the critical challenge is the pre‐leakage ahead of their reach to action site. Intuitively, a layer of coated expedient to cargo drug could alleviate the occurrence of unwanted drug release. Therefore, numerous nanoscale polymeric vehicles have been developed for anticancer drugs delivery to diseased sites. Most of these polymeric DDS are preferable to the physical encapsulation of drugs via the noncovalent interactions driven by the self‐assembly of amphiphilic polymers, leaving the potential risk untimely drugs leakage during their circulation. To address the challenge, drugs can be covalently attached to polymeric DDS and adapted as an inactive, masked prodrug, in which the controllable drug release can be obtained to respond tumor specific physiological environment.

### 3.1 Endogenous Stimuli‐Responsive Polymer‐Prodrug Conjugates

In early stage, the self‐assembling polymer‐prodrug conjugates (PDC) approaches depended upon enzymatic hydrolysis of amide linkage, a particularly ineffective way. Introducing of an effective mechanism to liberate the parent drug is, is hence indispensable. Currently, variety of stimuli cleavable bonds have been formed for use in polymeric prodrug delivery carriers, exemplified by pH‐, redox‐, and enzyme‐sensitive nanoprodrugs.

#### 3.1.1 pH Responsive

High even uncontrollable metabolic performance and insufficient diffusion lead to the accumulation of acidic metabolites in the tumor microenvironment. The relatively well‐maintained intracellular pH and the acidity of the interstitial space in tumors affect cancer cell function, mutual communication, and interactions with the extracellular matrix. Tumor pH is spatiotemporally heterogeneous, and the compartmentalized pH microenvironment favors cancer proliferation. Numerous researches showed the association between pH and cancer; Cancer booms in acidic environment, while barely survive in a normal, more alkaline milieu. It is documented that tumors develop extracellular microenvironments with pH ≈ 6.5–7.0 due to their abnormal metabolism in comparison with normal tissues, which provides an exploitable avenue to design smart pH oriented NP drug delivery systems for the cancer treatment,^[^ ^103^ ^]^ taking pH responsive polymer‐prodrug conjugates for example.

**Figure 8:**
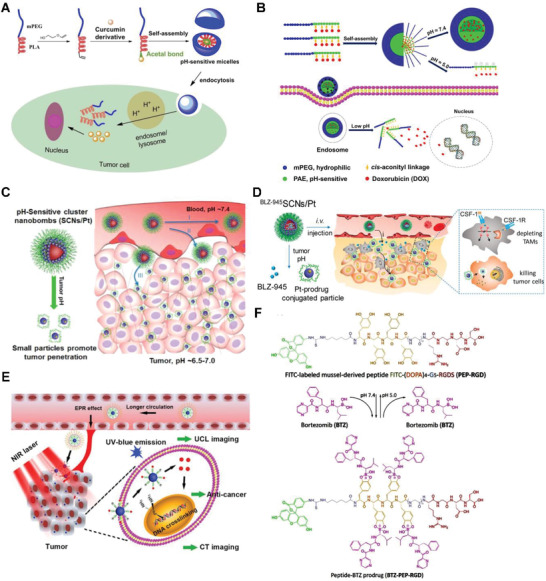
A) Illustration of acetal‐linked polymer‐CUR conjugate micelle and its endocytosis. Reproduced with permission.^[^ ^104^ ^]^ Copyright 2016, Elsevier. B) Schematic illustration of SCNs/Pt as a robust nanoplatform to overcome biological barriers to in vivo drug delivery in poorly permeably pancreatic tumor models. Reproduced with permission.^[^ ^105^ ^]^ Copyright 2018, Elsevier. C) Schematic illustration of SCNs/Pt as a robust nanoplatform to overcome biological barriers to in vivo drug delivery in poorly permeably pancreatic tumor models. Reproduced with permission.^[^ ^111^ ^]^ Copyright 2016, American Chemical Society. D) Schematic illustration showing the mechanism of spatial delivery of BLZ‐945 and Pt‐prodrug to TAMs and tumor cells. Reproduced with permission.^[^ ^114^ ^]^ Copyright 2017, American Chemical Society. E) Schematic illustration of CT/UCL dual‐modal imaging and NIR photon improved cancer therapy process for UCNs‐Pt(IV)@PEG‐PAH‐DMMA. Reproduced with permission.^[^ ^116^ ^]^ Copyright 2017, Elsevier. F) Structural formula of the mussel‐derived cancer‐cell‐targeting peptide and the dynamic conjugation of antitumor drug BTZ with pH‐responsiveness. Reproduced with permission.^[^ ^122^ ^]^ Copyright 2019, American Chemical Society.

To diversify the pH‐responsive CUR delivery platforms, CUR was conjugated to copolymer methoxy poly(ethylene glycol)‐poly(lactic acid) (mPEG‐PLA) via an acetal bond then assembled into pH‐labile nano‐micelles (**Figure** **8**A).^[^ ^104^ ^]^ DOX also conjugated to amphiphilic diblock copolymer poly (ethylene glycol) methyl ether‐*b*‐poly (*β*‐amino esters) through acid‐labile *cis* aconityl moiety (mPEG‐b‐PAE‐*cis*‐DOX) and assembled into polymeric micelles (PM) (Figure 8B); when treated with DOX *cis*‐PMs, the survival rate of mice is 30% higher than that of free DOX treated.^[^ ^105^ ^]^ Schiff base linkage between DOX and polylactide scaffold,^[^ ^106^ ^]^ hydroxyethyl starch^[^ ^107^ ^]^ also actualized acid‐triggered drug release profile and enhanced therapeutic efficiency comparing to DOX·HCl alone, so as to hydrazone bond bridged conjugate.^[^ ^108^ ^]^ An acid‐responsive star‐like polymeric prodrug that combined the therapeutic and diagnostic functionality in one unit.^[^ ^109^ ^]^ Etrych et al.’s investigation shown that both linear and star‐shaped polymer‐hydrazone linkage‐DOX prodrug based nanomedicine were able to inhibit aggressive lymphomas almost equally.^[^ ^110^ ^]^

To improve therapeutic efficacy of nanomedicines, remodeling of the tumor microenvironment, including tumor vessels normalization and the collagen matrix degradation have been reported. Alternatively, rationally regulating the particle shape and size of NPs provides other options. Given the observation that larger NPs remain high propensity to extravasate across tumor vasculature and gather in the locality of blood vessels, but hardly penetrate and distribute in the compressed tumor matrix. However, smaller NPs often readily penetrate the tumor, but often suffer from poorer circulating half‐life time in blood vessels and accumulation in tumor. Such dilemma calls for the development of a size‐changeable vesicle that keep large original size for prolonged vascular circulation and selective extravasation, while converting to small ones at tumor sites for deeper penetration and effective distribution. The stimuli to trigger vesicle sizes change is either exogenous or endogenous, Li et al. developed a vesicle in which platinum(Pt)‐prodrug dendrimers then self‐assembled into ultra‐pH sensitive cluster nanoparticles (SCNs); at neutral pH of blood, SCNs had an initial size of ≈80 nm while switch rapidly to less than 10 nm NPs in the mildly acidic tumor microenvironment (Figure 8C) therefore facilitate not only enhanced accumulation when NPs are big but also faster diffusion when they dissociated.^[^ ^111^ ^]^

Considerable investigations have demonstrated that the NPs’ physiochemical properties, particularly surface charge significantly impact their systemic transport in the body therefore many attentions have been paid on that. Nanoscale coordination polymers (NCPs) was formed by adding PEGylated poly‐L‐histidine into the mixture of metal ions and dicarboxylic cisplatin (IV) prodrug; NCPs exhibited efficient passive accumulation in the tumor where the mild acidic pH triggered charge conversion and size expansion hence enhance their tumor retention and cell uptake in vivo.^[^ ^112^ ^]^ Tumor acidity‐triggered ligand‐exposure NP were fabricated by integrating a Ce6‐modified acid‐responsive diblock copolymer and an iRGD‐modified polymeric DOX prodrug.^[^ ^113^ ^]^ Tumor microenvironments (TAMs) and tumor cells are two subpopulations in tumor tissues. TAMs widely distributed in well‐perfused areas to improve tumor proliferation, while tumor cells spread throughout the tumor mass in bulk. To potentiate the drug therapeutic efficacy and eradicate tumor tissue, delivering multiple therapeutic drugs to both TAMs and tumor cells in one compartment is appealing. This idea was implemented by further development of previous SCNs, in which TAM inhibitor BLZ‐945 was physically encapsulated and Pt prodrug was covalently conjugated in the hydrophobic part of NPs (Figure 8D), then functioned in different area of tumor tissues; TAMs preferentially taken up the released BLZ‐945 cause their depletion while the small Pt‐prodrug carrying particles enable deep tumor penetration, this concurrent chemoimmunotherapy, therefore was more effective in multiple tumor models in comparison with its monotherapy peer.^[^ ^114^ ^]^ Similarly, a polymeric prodrug of DOX, a photosensitizer and a pH‐responsive di‐block copolymer formed micelles converted as active form in weakly acid conditions and displayed a 7.5‐fold of increase of fluorescence, comparing to at pH 7.4.^[^ ^115^ ^]^ Xu et al. constructed a Pt(IV) prodrug based nanoplatform, loaded NaY‐F4:Yb,Tm upconversion NPs then coated with polymer, can response to the mild acidic stimulus (pH ≈6.5) of tumor extracellular microenvironment and experience charge‐shifting to a cationic, leading to efficacious cell internalization due to strong electrostatic attraction negative cell membrane (Figure 8E).^[^ ^116^ ^]^ Ma and co‐workers developed a DOX‐conjugated polymeric prodrug, which could self‐assemble into shell‐core structural micelles with pH‐triggered charge conversion from negative to positive.^[^ ^117^ ^]^

Bortezomib (BTZ), written as Pyz‐Phe‐boroLeu, is a N‐protected dipeptide boronic acid analogue that inhibits cancer cell proteasome through covalent bond formation between its boronic acid moiety and the residues of Serine 26 in the catalytic site of several proteases.^[^ ^118^ ^]^ Nevertheless, the lack of therapeutic effects was still observed both when BTZ was used alone and it was combined with other agents.^[^ ^119^ ^]^ An important characteristic of the boronic acid‐diol^[^ ^120^ ^]^ complexes (B‐complexes) is formed through dynamic covalent chemistry and is reversible in a pH‐dependent manner. At neutral or alkaline pH, B‐complexes are in stable state so the BTZ is deactivated; in a low‐pH environment, the B‐complexes readily dissociates to release free toxic BTZ. This mechanism can be potentially exploited for both localized extracellular drug release within the mildly acidic tumor interstitium and intracellularly in the more acidic endosomes. In both cases, the acidic environment would trigger BTZ dissociation, inhibiting proteasome activity.^[^ ^121^ ^]^ Currently, the widely studied antitumor drug nanocarriers are xenobiotic, including synthetic polymers, inorganic NPs or their hybrids, hence may related with unpredictable harmness to lives. Accordingly, Ma and co‐authors prepared a nanocarrier based on the self‐assembly of biomimetic peptides with BTZ through the dynamically conjugation of the catechol groups of the peptide with the boric acid moiety (Figure 8F); this pH‐sensitive conjugates released ≈80% of the BTZ within 12 h at pH 5.0, while less than 20% drug was liberated in the same term.^[^ ^122^ ^]^ Dissimilar to conventional nanoprodrug designs, which often require bioconversion of liver, molecularly imprinted polymer (MIP)‐based prodrug delivery is liver‐independent. Using boronate affinity controllable oriented surface imprinting approach,^[^ ^123^ ^]^ Gu et al. developed an MIP oriented prodrug delivery system using sialic acid and 5’‐deoxy‐5‐fluorocytidine as co‐templates and fulfilled specifically accumulated at the tumor site and sustainably released with tumor‐dependent manner.^[^ ^124^ ^]^

It was often observed that monotherapy for cancer through a single mechanism showed limited efficacies due to drug‐resistant mutations. Alternatively, drug combinations with different pathway can synergically suppress cancer proliferation and minimize the development of drug resistance. To synchronize pharmacokinetic profiles and synergize activities of BTZ and DOX in solid tumor treatment, micellar monodispersed NPs were constructed by a spatially segregated, linear‐dendritic telodendrimer containing two parts: a hydrophilic PEG corona, a hydrophobic interior consisting of bortezomib catechol/diol prodrug conjugate,^[^ ^125^ ^]^ and a dendritic DOX‐Rh composite through *π*–*π* stacking. Together with alendronate, BTZ‐catechol conjugated prodrug made of nanomedicines fulfilled bone‐targeting delivery of chemotherapeutics.^[^ ^126^ ^]^ Hu et al. developed NPs from an amphiphilic PEGylated dendrimer with dopamine and was able to form the conjugate with BTZ, this interaction was weakened at low pH, consequently ensured the constant release of BTZ in the acidic environment of tumor tissues.^[^ ^127^ ^]^

#### 3.1.2 Redox Responsive

In tumor, the presence of reactive oxygen species (ROS) is remarkably higher than that in normal ones; significant difference in GSH concentration existed between the intra‐ and extracellular domain (mM vs µM) of tumor tissues, such heterogeneous ROS and/or GSH distribution offer an opportunity to develop redox responsive drug delivery system therefore was frequently explored.

**Figure 9:**
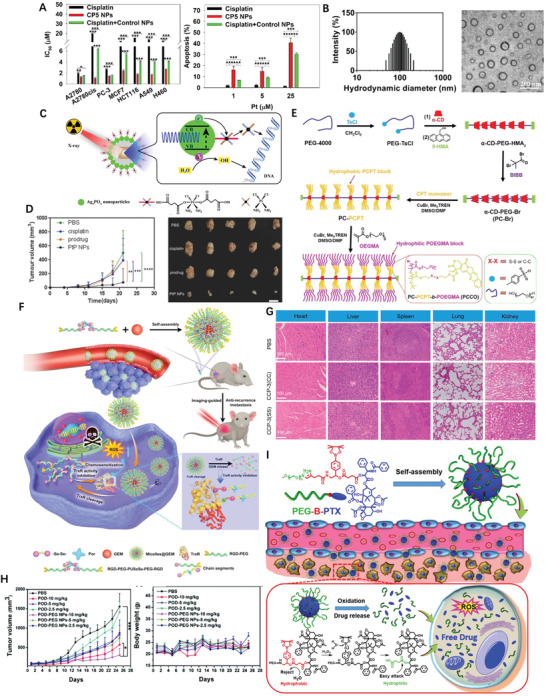
A) In vitro cytotoxicity and apoptosis of cells treated with cisplatin, CP5 NPs, or cisplatin + control NPs. Reproduced with permission.^[^ ^128^ ^]^ Copyright 2018, American Chemical Society. B) Histogram of particle‐size distribution of P6 NPs obtained by DLS and TEM morphologies of P6 NPs stored in water. Reproduced with permission.^[^ ^129^ ^]^ Copyright 2019, American Chemical Society. C) Diagram showing the Preparation Process and Mechanisms Underlying the Effects of X‐PDT with LiLuF4:Ce@SiO_2_@Ag_3_PO_4_@Pt(IV) NPs. Reproduced with permission.^[^ ^133^ ^]^ Copyright 2018, American Chemical Society. D) In vivo bioluminescence images of mice bearing luciferase PC3 cell xenograft tumors post 10 d treatment with different groups. And resected luciferase‐expressing PC3 tumors from experimental groups at the 21st day. Reproduced with permission.^[^ ^134^ ^]^ Copyright 2017, Wiley‐VCH. E) Schematic illustration of the preparation of amphiphilic PCCO prodrug. Reproduced with permission.^[^ ^135^ ^]^ Copyright 2018, Elsevier. F) Schematic illustration of TrxR‐responsive DDS for ROS‐mediated chemosensitization and anti‐recurrence/metastasis therapy based on TrxR activity inhibition. Reproduced with permission.^[^ ^144^ ^]^ Copyright 2020, Elsevier. G) H&E staining images for major organs of MCF‐7 tumor‐bearing nude mice after different treatments for 15 days. Reproduced with permission.^[^ ^149^ ^]^ Copyright 2019, American Chemical Society. H) ROS responsive polymer‐drug conjugates in vitro In vivo antitumor efficacy and body weigh after intravenous injection of different drug formulations (with POD‐equivalent dose of 2.5, 5, and 10 mg kg^−1^, respectively). Reproduced with permission.^[^ ^150^ ^]^ Copyright 2019, Royal Society of Chemistry. I) Self‐assembly of MPP NPs and the ROS generation process. Reproduced with permission.^[^ ^155^ ^]^ Copyright 2020, Elsevier.

Having known the cisplatin resistance in tumor cells is positively correlated with increased levels of intracellular GSH, Ling et al. introduced high density disulfide bond into the polymer backbone then encapsulated cisplatin prodrug into NPs with average diameters of 76.2 nm, sharp response to GSH, and notable apoptosis of cisplatin‐resistant cancer cells (**Figure** **9**A).^[^ ^128^ ^]^ Continuously, multiple Pt(IV) prodrugs were designed by precisely tuning axial ligands, followed by self‐assembling with lipid‐PEG to form redox‐responsive Pt(IV) NPs; the optimized one possessed particle size of 99.3 nm (Figure 9B), Pt loading of 11.24%, remained stable within a week, and swift redox‐triggered release.^[^ ^129^ ^]^ The prodrug micelles consisting of PTX and D‐*α*‐Tocopheryl polyethylene glycol with cleavable disulfide bond showed 55% and 91% more effective than that of Taxol and uncleavable prodrug, respectively.^[^ ^130^ ^]^

NPs containing cisplatin prodrug (CPP) and DTX showed an additive therapeutic effects in treatment efficacy versus the free drug combination in vivo.^[^ ^131^ ^]^ The histone deacetylases inhibitor vorinostat was co‐loaded with CPP into synthetic polymer for enhancement of DNA lesion formation and prevention of the subsequent repair at the same time.^[^ ^132^ ^]^ The CPP was also utilized as a sacrificial electron acceptor fluorescence produced by nanoscintillators to increase the hydroxyl radicals (·OH) yield, therefore enhanced the curative effects of X‐ray‐induced‐PDT (Figure 9C).^[^ ^133^ ^]^ By cross‐linking with Fe^3+^ ions, CPP‐polyphenol and low‐fouling PEG‐polyphenol derivatives were co‐assembled into nanocomplexes which displayed four times higher inhibition against tumor growth than cisplatin (Figure 9D).^[^ ^134^ ^]^ CD polyrotaxanes branched hydrophilic PEG chain and hydrophobic polymeric camptothecin (CPT) chain formed nano‐sized polymeric micelles (Figure 9E), which could release more than 85% of the payload via GSH triggered break‐down of the disulfide linker.^[^ ^135^ ^]^ Tumor microenvironment was reshaped by prodrug NPs via the amplification of the tumor oxidative stress and simultaneously actualize ROS‐responsive CPT release, in which the drug was linked to polymer through ROS‐responsive thioketal (TK) bond.^[^ ^136^ ^]^ CPT and photosensitizer were concurrently conjugated to the polymer via TK linkage;^[^ ^137^ ^]^ In another case, TK bond connected CPT‐photosensitizer conjugate prodrug NPs, together with platinum nanozyme were co‐PEGylated into one unit which catalyzed the decomposition of H_2_O_2_ to produce O_2_ for alleviate hypoxia in tumor tissue and enhanced PDT efficiency.^[^ ^138^ ^]^ TK bridged PTX dimer and photosensitizer were co‐camouflaged with red blood cell membrane into prodrug NPs which easily internalized endosomes hence actualized not only PDT but also triggered PTX_2_‐TK cleavage related chemotherapy.^[^ ^139^ ^]^ To pass the blood brain barrier and target glioma cells, internalizing RGD peptide was anchored to nanosized polymeric micelles of CPT‐S‐S‐polyethylene glycol (PEG).^[^ ^140^ ^]^ Although the co‐delivery of photosensitizers with ROS sensitive prodrugs for light triggered ROS production and programmable prodrug activation are striking, it remained a challenge to precisely tune the ratio of the two components. Chen et al. hence developed a supramolecular platform with the optimized loading ratio between the photosensitizer and prodrug by the means of host‐guest strategy and maximized combination therapy efficacy in the cancer treatment.^[^ ^141^ ^]^

Most carriers of polymer‐drug conjugate platforms for drug delivery have no therapeutic activities, yet seemed economically inefficient because the major components of formulated drug by mass are excipients or carriers. Therefore, it might be more rational to design the polymer with biomedical functions. Selenium, for instance, has been introduced into polymeric chain;^[^ ^142^ ^]^ the synthetic routes of selenium‐containing polymers, specific stimuli‐responsive properties together with diverse applications have been summarized.^[^ ^143^ ^]^ Recently, The diselenium‐containing triblock polymer, developed by Xu's group, self‐assembled as micelles for imaging‐guided GEM chemosensitization and anti‐recurrence/metastasis therapy; notably, chain segments of the polymer targeted the active site of thioredoxin reductase via Se‐Se/Se‐S exchange reactions for activity inhibition hence full drug delivery composite (Figure 9F) for therapeutic.^[144]^ In terms of the physical stability and biocompatibility, lipid‐polymer NPs (LPNs) exhibit complementary features of both polymeric and lipidic NPs. Wang et al., hence developed a nanoplatform by conjugation of RGD peptide‐contained redox‐sensitive PTX prodrug with lipid‐polymer to give LPNs that suppressed the tumor size from 1486 to 263 mm^3^, more than 82% of shrinking.^[^ ^145^ ^]^ PTX was grafted onto amphiphilic polymer through click reaction to give a reduction‐sensitive polymeric prodrug, then co‐assembly with red aggregation‐induced emission fluorogen as micelles, achieved image‐guided PDT and chemotherapy.^[^ ^146^ ^]^ A star‐like polymeric prodrug micelles of Ir was prepared and presented reduction‐active drug release pattern.^[^ ^147^ ^]^ To treatment efficacy of NO, GSH‐sensitive NO donor coordinates with ions to form the nanoscale coordination polymer (NCP) via precipitation and then partial ion exchange process; beside quick NO release triggered by high concentrations of GSH in tumor cells, under high concentrations of H_2_O_2_ in tumors, the NCP fulfilled Fenton reaction to generate toxic ·OH hence got the diseased tissues damaged.^[^ ^148^ ^]^

One of challenges in the supramolecular assemblies of amphiphilic prodrugs is to reduce/avoid the dissociation risk provoked by biological environment change before reaching the tumor tissues, attributing to affected fidelity of assembly by disturbed hydrophobic‐hydrophilic balance. Shi and co‐worker constructed unimolecular micelles (UMs) based starburst polyprodrug consisting of linear diblock copolymers with polymerized reduction‐responsive CPT as hydrophobic part and PEG as hydrophilic part;^[149]^ by adjusting amphiphilic block ratio, several copolymeric prodrugs with different drug up to 25% loading and various sizes around 30 nm UMs with excellent micellar stability were obtained, together with improved therapeutic efficacy and minimal side effect for in vitro and in vivo cancer therapy (Figure 9G).^[^ ^149^ ^]^

It is also known the ROS level in tumor microenvironment is hundreds of times higher than that of in normal tissues, providing an opportunity for oxidation sensitive drug release. Ou et al. linked anticancer agent podophyllotoxin (POD) to PEG through a H_2_O_2_‐responsive oxalate ester bond, thus allowed effective POD release; effective antitumor activity (Figure 9H) was observed against colon carcinoma CT26 cells and CT26 tumor‐bearing mice.^[^ ^150^ ^]^ Short half‐life in blood of the manganese porphyrin (MnP) heavily limited its effective treatment for acute liver failure, Ko et al. therefore developed MnP loaded antioxidant polymeric prodrug poly(vanillyl alcohol‐co‐oxalate) particles which enable the H_2_O_2_ sacrifice and anti‐inflammatory activities in macrophages.^[^ ^151^ ^]^ CPT prodrug‐based nanoreactor with glucose oxidase (GOD) encapsulated offered the high‐efficiency in situ CPT transformation and the production of high concentration H_2_O_2_, therefore synergistically killed cancer cells due to high tumor oxidative stress and chemotoxicity.^[^ ^152^ ^]^ The H_2_O_2_ was converted further into the highly toxic hypochlorous acid.^[^ ^153^ ^]^ A reduction‐responsive heterodimer of photosensitizer pheophorbide A (PPa) and immunotherapy agent NLG919, and a light‐activatable prodrug of oxaliplatin were coencapsulated into one nanoplatform which inhibit the tumor growth, lung metastasis, and tumor recurrence.^[^ ^154^ ^]^ The conjugation of PTX with others generally occurred at its 2′‐hydroxyl group via the esterification reaction, but the ester is often too stable to release the drug in the cytosol. A *p*‐(boronic ester)benzyl‐based tumor‐specifically cleavable linker was introduce to bridge PTX and low molecular weight PEG (Figure 9I); the obtained conjugate self‐assembled particles with ≈50 nm in size were stable at the normal physiological environment, but fast liberated and released PTX in response to the raised ROS level in tumors.^[^ ^155^ ^]^

#### 3.1.3 Enzyme Responsive

Tumor‐associated enzyme‐activated prodrug is a class of chemotherapeutics that can be specifically activated within treated tumors where the enzyme is overexpressed. Overexpressed glutathione transferases (GSTs) are frequently associated with bad prognosis and anticancer drug resistance, so DOX prodrug with sulfonamide and acetyl moiety incorporated were developed.^[^ ^156^ ^]^

The passive diffusion of nanomedicines to tumor microenvironment is generally believed to be difficult due to the particular characteristics of tumor angiogenesis.^[^ ^157^, ^158^ ^]^ Hence, actively transport nanomedicines across the capillary wall into tumor tissues is of great interesting.

**Figure 10:**
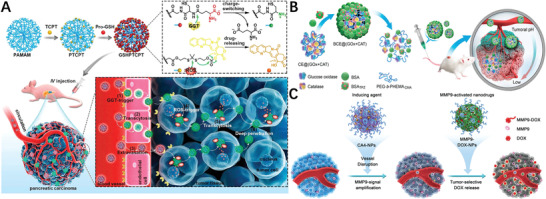
A) Schematic representation of GGT‐triggered transcytosis of the dendrimer‐camptothecin conjugate for PDA therapy. Reproduced with permission.^[^ ^161^ ^]^ Copyright 2020, American Chemical Society. B) Schematic showing the nanoclustered cascaded enzymes for targeted tumor starvation and deoxygenation‐activated chemotherapy without causing systemic toxicity. Reproduced with permission.^[^ ^163^ ^]^ Copyright 2019, American Chemical Society. C) Illustration of cooperative cancer treatment by combining CA4 nanodrug plus MMP9‐activated DOX prodrug nanomedicine. Reproduced with permission.^[^ ^167^ ^]^ Copyright 2019, Wiley‐VCH.

Membrane *γ*‐glutamyl transpeptidase (GGT), overexpressed on in human tumors, can cleave *γ*‐glutamylamides and has been related to the treatment of acute renal injury/failure using *N*‐hydroxyguanidine prodrugs.^[^ ^159^ ^]^ Zhou and co‐authors therefore prepared a zwitterionic polymer‐CPT conjugate enabling the GGT‐mediated cationization, which enabled active endocytosis and transcytosis and uniform distribution throughout the tumor tissues.^[^ ^160^ ^]^ The dense fibrotic stroma in pancreatic ductal adenocarcinoma (PDA) prevented drug diffusion from the tumor. Thereby, Wang et al. developed the GGT‐triggered charge‐reversal dendrimer‐drug nano conjugate (**Figure** **10**A) by attaching CPT prodrug to polyamidoamine dendrimers with penetration‐promoting properties mainly through cell endocytosis and transcytosis process.^[^ ^161^ ^]^

Due to extremely vigorous glucose metabolism of cancer cells comparing to normal ones, intratumoral glucose depletion‐based cancer starvation represents a novel strategy for cancer therapy, but it is often compromised by concurrent toxicity to normal cells since they survived from the glucose aerobic glycolysis; cancer cells could bypass the well understood glucose metabolic pathway due to metabolic plasticity^[^ ^162^ ^]^ therefore lead to the resistance. Aside from the indirect blocking the glucose pathways, direct clearance of intratumoral glucose is of particular consideration in cancer therapy since cancer cells are more sensitive to glucose level fluctuation. Ma et al. proposed a cascaded catalytic nanomedicine by combining the glucose‐depletion therapy and deoxygenation‐activated prodrug (Figure 10B), in which both glucose molecules and O_2_ molecule were consumed simultaneously therefore led to cancer cell death from starvation.^[^ ^163^ ^]^

To address the challenge of antimetastasis therapy, the prodrug of cytotoxic soravtansine (DM4) and legumain‐specific propeptide of melittin was anchored on membrane of the living macrophages to fabricate bioengineered a nano delivery system which was activated by legumain protease to release DM4 and displayed superior targeting efficiency for lung metastasis.^[^ ^164^ ^]^ Matrix metalloproteinase‐2 (MMP‐2) is often overexpressed in most solid tumors to promote metastasis and invasion, hence Gap et al. constructed the MMP‐2‐liable peptide spacer incorporated prodrug vesicles which amplified antitumor immunity to eradicate the tumor in two breast immunocompetent mouse models.^[^ ^165^ ^]^ The self‐delivery NPs was developed by conjugating programmed cell death ligand 1 (PD‐L1) inhibitor Metformin with photosensitizer Ce6 through MMP‐2 cleavable peptide GPLGVRGDK, fulfilled superior targeting ability, hampered the PD‐L1 expression in breast cancer tissue.^[^ ^166^ ^]^ Improvement of the chemotherapeutics selectivity through tumor‐associated enzyme‐activated prodrugs is appealing strategy. However, not all tumor‐associated enzymes are always expressed in large quantity. As a result, the shortage but are essential for prodrug activation of these enzymes usually compromises the antitumor potency. A striking approach was to cooperate Combretastatin A4 (CA4) nanodrug and matrix metalloproteinase 9 (MMP9)‐activated DOX prodrug NPs (Figure 10C); the prior use of CA4 aggravated the tumor hypoxia state, sequentially enhanced MMP9 expression by 5.6‐fold in tumors, hence enhance tumor‐selective active drug release by 3.7‐fold in animal model.^[^ ^167^ ^]^

### 3.2 Exogenous Stimuli‐Responsive Polymer‐Prodrug Conjugates

#### 3.2.1 Light Stimuli‐Responsive

**Figure 11:**
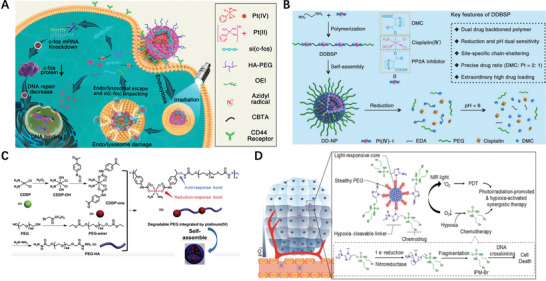
A) Schematic illustration of the photoactivatable Pt(IV) prodrug‐backboned polymeric nanoparticle system (CNPPtCP/si(c‐fos)) for light‐controlled efficient gene delivery and synergistic PACT and RNAi on platinum‐resistant ovarian cancer. Reproduced with permission.^[^ ^171^ ^]^ Copyright 2020, American Chemical Society. B) Schematic illustration of dual drug backboned shattering polymeric theranostic nanomedicine (DDBSP) for synergistic eradication of patient‐derived lung cancer. Reproduced with permission.^[^ ^174^ ^]^ Copyright 2018, Wiley‐VCH. C) Synthetic routes and scheme of degradable PEG integrated by platinum for cancer treatment. Reproduced with permission.^[^ ^175^ ^]^ Copyright 2019, Elsevier. D) Schematic of SPNpd for hypoxia‐activated synergistic PDT and chemotherapy. Reproduced with permission.^[^ ^177^ ^]^ Copyright 2019, Wiley‐VCH.

Photochemistry provides an unique option for precise therapy because not any additional reagents required,^[^ ^168^ ^]^ only light. Recently, more and more light triggered components have been integrated into polymer‐prodrug conjugates platform. In most of block copolymer (BCP) drug delivery system, drugs are often grafted to polymer main chain through liable bonds and embed in the hydrophobic cores of BCP polymeric particles.^[^ ^169^ ^]^ Alternatively the drug moieties can also be integrated through repetitive photo‐cleavable moieties such as UV‐sensitive *ο*‐nitrobenzyloxy‐l‐carbonyl^[^ ^170^ ^]^ into the main chains of BCPs. A photoactivatable Pt(IV) prodrug‐backboned polymeric NP was reported by Zhang et al.; when blue‐light (430 nm) applied, the NP produced oxygen‐independent N_3_^•^ in a controlled manner to induce efficient endo/lysosomal escape (**Figure** **11**A).^[^ ^171^ ^]^

These photo‐breakable group‐backboned BCP particles disassembled faster than the ones with single photo‐cleavable joint within amphiphilic component or those with multiple cleavable side groups on hydrophobic blocks when they were exposed to UV light. However, the above approach often needs photochromic whose by‐products are toxic and the option for light source was limited. The restrategization of integrated photo‐sensitive Pt(IV)‐azide prodrugs into polymer main chain and self‐assembled into particles which can be photo‐activated from UV to visible light.^[^ ^172^ ^]^ Additionally, similar pH/redox dual‐responsive backboned BCP prodrug platform was constructed by He et al. and utilized to co‐delivery DOX.^[^ ^173^ ^]^ A dual drug demethylcantharidin (DMC) and cisplatin backboned shattering polymer was proposed, in which cisplatin shouldered DMC in two sides was synthesized at first, then polymerized with ethylenediamine and self‐assembled into NPs[174] (Figure 11B). The stability of high molecular weight PEG is beneficial for drug encapsulation and delivery, but may cause side effects if the PEG residual accumulated in the body. Hence, Qian et al. designed polymeric drug delivery system consisting of low molecular weight PEG and platinum (IV) prodrug with responsive bonds in the polymeric backbone[175] (Figure 11C). NP comprised of polysilsesquioxane crosslinked by a cisplatin prodrug with drug loading of 42% showed higher therapeutic efficacy than cisplatin alone.^[^ ^176^ ^]^ Although photodynamic therapy (PDT) represents a promising noninvasive approach toward cancer therapy, its efficacy is often deteriorate due to tumor hypoxia. Nevertheless, hypoxic features of cancer microenvironments may favor tumor treatments. Amphiphilic polymer brush, comprised light‐responsive photodynamic backbone conjugated with DNA alkylator through hypoxia‐cleavable linkers, self‐assembled into nanoprodrug which exerted synergistic PDT and chemotherapy (Figure 11D), and effectively inhibited tumor growth in animal model.^[^ ^177^ ^]^ Anticancer drugs CUR and mitoxantrone backboned and reduction‐sensitive polyprodrug at the predefined ratio release drug with a synergistic anticancer effect was demonstrated by in vitro and in vivo experiments.^[^ ^178^ ^]^

#### 3.2.2 Ultrasound Stimuli Responsive

**Figure 12:**
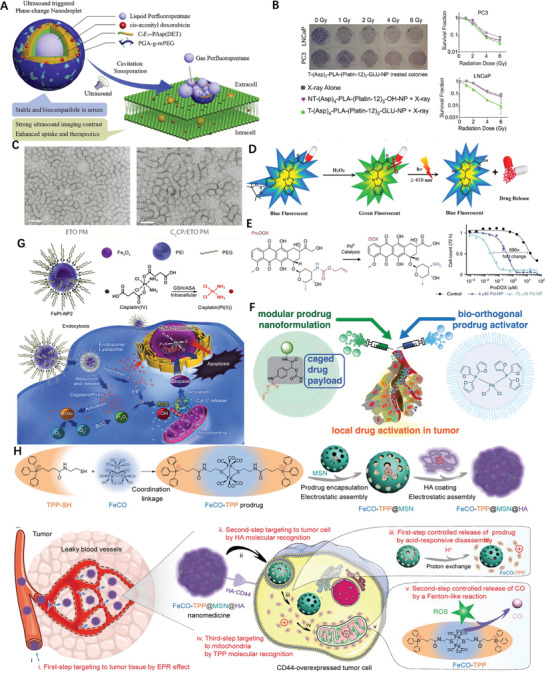
A) The mechanisms of ultrasound‐triggered imaging and delivery of CAD. Reproduced with permission.^[^ ^179^ ^]^ Copyright 2019, Elsevier. B) Activity of combination therapeutic nanoparticle in combination with radiation) TEM images of as‐prepared micelle, Scale bar = 100 nm. Reproduced with permission.^[^ ^181^ ^]^ Copyright 2018, Elsevier. C) TEM images of worm‐like polymeric micelles. Scale bar = 100 nm. Reproduced with permission.^[^ ^183^ ^]^ Copyright 2018, American Chemical Society. D) Working protocol of activatable nano prodrug. Reproduced with permission.^[^ ^184^ ^]^ Copyright 2017, American Chemical Society. E) DOX prodrug being converted by Pd into uncaged DOX through allylcarbamate cleavage and Pd‐NP cotreatment enhances the concentration of proDOX causing 50% reduction in cell count. Reproduced with permission.^[^ ^187^ ^]^ Copyright 2017, Nature Publishing Group. F) Overview of modular prodrug design strategy: a self‐immolative linker (gray) bridges three modular functional aspects of an inactive nontoxic prodrug. Reproducedunder the terms of the Creative Commons CC‐BY license.^[^ ^188^ ^]^ Copyright 2018, American Chemical Society. G) Construction of self‐sacrificing iron oxide NPs with cisplatin(IV) prodrug (FePt‐NP2) circumvents the endocytosis of cisplatin into the cells. Reproduced with permission.^[^ ^189^ ^]^ Copyright 2017, American Chemical Society. H) Schematic illustration of multistage assembly method for construction of the FeCO‐TPP@MSN@HA nanomedicine. Reproduced with permission.^[^ ^190^ ^]^ Copyright 2020, American Association for the Advancement of Science.

Ultrasound (US), an exterior stimulus, is prior to many other physiochemical stimuli with its decent patient acceptance, in depth triggering, cost efficiency, noninvasiveness and spatiotemporal regulation. So Gao et al. integrated US responsive phase‐change contrast agent, acid‐cleavable DOX prodrug and cationic amphiphilic fluorinated polymer to construct a nanodroplet which facilitated the US contrast imaging and drug delivery simultaneously (**Figure** **12**A).^[^ ^179^ ^]^ Recent report showed that US‐induced central bond scission could also activate the CPT release from inactive macromolecules through the mechanochemical manner.^[^ ^180^ ^]^ Beside chemotherapy, many other treatment choices exist for non‐metastatic and metastatic advanced stage cancer including radiation therapy. Some cancers, such as castration‐resistant prostate cancer is accompanied with inflammatory processes, so anti‐inflammatory agents should also play an important role in cancer suppression. Therefore, Pathak et al. combined an engineered prostate‐specific membrane antigen targeted polymer‐based self‐assembled NP platform integrating a Pt prodrug and aspirin prodrug; after irradiation, the nano‐system tuned the mitochondrial metabolism, cellular inhalation and the DNA damage (Figure 12B).^[^ ^181^ ^]^ To improve stability and achieve sustained drug release, stereo‐complex prodrugs of oligo(lactic acid)_10_‐GEM was incorporated into PEG‐*b*‐PLA micelles to achieved *t*_1/2_ of 60 h.^[^ ^182^ ^]^ Although co‐delivery of multiple drugs nanoplatform can improve cancer inhibition, faces challenges because of different solubility for drug incorporation. A worm‐like polymeric micelle (Figure 12C) based on amphiphilic block copolymer together with cisplatin prodrug enabled co‐formulation of etoposide with wide range of drug mixing ratios and remarkable high drug loading of over 50% wt.^[^ ^183^ ^]^ Nanoprodrug activatable nano prodrug pinpointed the tumor sites by fluorescent color change using boronate fluorophore, followed by precise spatiotemporal irradiation of light (Figure 12D) only on identified tumor sites to release of anticancer drug chlorambucil.^[^ ^184^ ^]^ It is believed photothermal therapy (PTT) represents an appealing alternative/supplement to traditional cancer therapies mainly due to its non‐invasive and controllable features. Given the observation of co‐occurrence of tumor and inflammation, Dong et al. proposed a strategy whereby gold nanorod‐encapsulated graphitic nanocapsule was combined with prodrug pyrene‐aspirin to both ablate solid tumor and simultaneously eliminate the inflammation when PTT was applied.^[^ ^185^ ^]^

#### 3.2.3 Bioorthogonal Chemistry Based and Gaseous ProDNMs

Metal‐encapsulated NPs have been developed to unravel the reactivity of abiotic metals in bioorthogonal chemistry. Recently, strategies for the delivery of such catalysts made of palladium to specific cell have been established.^[^ ^186^ ^]^ When Pd‐ and DOX prodrug NP were sequentially injected in vivo, nanoencapsulated Pd can be delivered to tumors and catalyze the conversion of the non‐toxic prodrugs to more cytotoxic DOX itself in a spatiotemporally (Figure 12E) constrained propensity.^[^ ^187^ ^]^ Further on, Miller et al. designed a modular prodrug nanoformulation platform that combined biorthogonal cleavage with a self‐immolative linker and an aliphatic anchor (Figure 12F), then leveraged the ability of Pd‐NP to trigger release of the activated drug to obtained >10^4^‐fold increased cytotoxicity upon prodrug activation.^[^ ^188^ ^]^ Having known that cisplatin mediated the production of H_2_O_2_ in cell, while iron catalyzed Fenton chemistry to turn H_2_O_2_ into ^•^OH which cause oxidative damages to cellular components, Ma et al. developed iron oxide self‐sacrificing nanocarrier with cisplatin(IV) prodrug and enhanced a Pt and Fe accumulation of 10.5‐ and 9.3‐fold via magnetic‐field mediated‐localization delivery respectively, comparing to monotherapy in animal model (Figure 12G).^[^ ^189^ ^]^

Some gaseous signaling molecules, such as carbon monoxide (CO) can selectively induce the cancer cells apoptosis and also protect normal cells when its concentration is well‐controlled. However, the diffusibility of CO after transmembrane is often targetless due to small molecule size. Currently, uptake of CO prodrugs with tumor‐targeting capabilities is one of main important strategies. In particular navigating the CO prodrugs to mitochondria was very effective since they can inhibit the mitochondrial aerobic respiration. To obtain a prodrug who enable both mitochondria accumulate in a targeted way and release CO in mitochondria in a responsive way, Meng and co‐workers therefore, constructed a mesoporous silica nanoparticle (MSN)‐based prodrugs Fe_3_CO_12_ which fulfilled the tumor tissue‐, cell‐ and mitochondria‐targeted delivery of the CO, consequentially the acid‐responsive release of CO from prodrug and the intramitochondrial reactive ROS‐responsive release of CO by Fenton‐like reaction (Figure 12H) both in vitro and in vivo.^[^ ^190^ ^]^ Although metal carbonyl complexes can easily liberate CO in response to activation stimulus, the use is limited because they are unstable under ambient conditions of oxygen or moisture. Wang et al. therefore encapsulated iron pentacarbonyl into the cavity of a Au nanocage which exhibits excellent biocompatibility, stability, safety, can be activated by NIR irradiation within the tumor environment to generate CO.^[^ ^191^ ^]^

### 3.3 Multi‐Stimuli Responsive Polymer‐Prodrug Conjugates ProDNMs

Although the mono‐stimuli‐responsive polymeric prodrug NPs pave the way to on‐demand drug delivery and release for cancer treatments, these monostimuli‐responsive DDS therapeutically perform unsatisfied due to the low tumor specificity or insufficient activation of the antitumor immune response. As previously mentioned, low pH, high GSH and unnormally expressed receptor on membrane are the key features of cancer cells.

**Figure 13:**
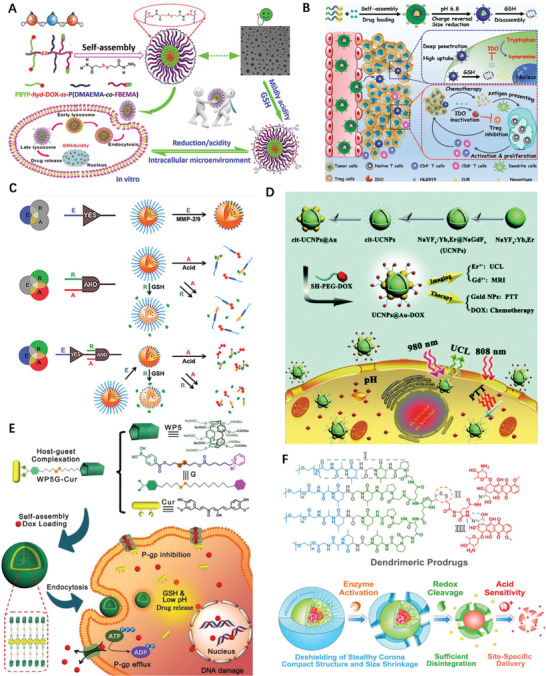
A) pH/redox responsive polymeric prodrug and fabrication of shell cross‐linked prodrug micelles for antitumor drug transportation. Reproduced with permission.^[^ ^192^ ^]^ Copyright 2018, American Chemical Society. B) Illustration of size‐shrinkable and charge‐reversal system for tumor chemo‐immunotherapy in vivo. Reproduced with permission.^[^ ^194^ ^]^ Copyright 2020, Elsevier. C) Construction of Boolean logic nanoplatform for combination immunotherapy of cancer. Reproduced with permission.^[^ ^195^ ^]^ Copyright 2020, Wiley‐VCH. D) Schematic illustration of the synthesis, upconversion luminescence magnetic resonance imaging, intracellular chemotherapy, and photothermal therapy (PTT) of UCNPs@Au‐DOX nanocomposites. Reproduced with permission.^[^ ^196^ ^]^ Copyright 2017, Royal Society of Chemistry. E) Schematic illustration of constructing bifunctional supramolecular prodrug NPs and their application in cancer therapy. Reproduced with permission.^[^ ^197^ ^]^ Copyright 2020, Royal Society of Chemistry. F) Schematic illustrations of molecular and supramolecular engineering on tumor‐specific multiple stimuli‐activated dendrimeric nanoassemblies with metabolic blockade and their synergistic effects for overcoming physiological barriers and cellular factors of chemotherapy resistance. Reproduced with permission.^[^ ^199^ ^]^ Copyright 2017, American Chemical Society.

In most cases, acidicidity and reductivity are often concurrently occurred in cancer tissues hence the construction of dual pH/redox responsive nanoprodrug platform seems advantageous than mono one.^[^ ^101^ ^]^ Li et al. fabricated a shell cross‐linked polymeric prodrug micelles; once the internalization of the prodrug micelles into the cancer cells, acylhydrazone bonds were cleaved under endosomal pH to release DOX and disulfide linkages broken to response high level GSH (**Figure** **13**A) of the cytosol individually, leading to the disassembly of prodrug micelles.^[^ ^192^ ^]^ Another pH/redox dual‐sensitive PEG composed of polymeric prodrug micelle enhanced chemotherapy effect on the tumor site while reducing the physiological toxicity of DOX.^[^ ^193^ ^]^ Immune checkpoint inhibitor NLG919 and tumor immunogenicity inducer CUR were co‐loaded into a nanosystem which is dual pH/redox‐responsive size‐shrinkable and charge‐reversal (Figure 13B) for chemotherapy‐improved immunotherapy.^[^ ^194^ ^]^ To further improve tumor‐specific delivery of the therapeutics, biologically stimuli‐responsive linkers, the enzymatically degradable peptide sequence, disulfide and ultra‐pH‐sensitive (Figure 13C) bonds were incorporated into the polymer chains to construct Boolean logic nanoplatform.^[^ ^195^ ^]^ A multifunctional cancer therapy nanocomposite was fabricated by linking the hydrazone bond bearing prodrug SH‐PEG‐DOX onto gold nanocrystals grown in situ on the surface of upconversion NPs (Figure 13D), consequently actualized pH‐responsive drug release and efficient PTT treatment in vitro under irradiation with an 808 nm laser.^[^ ^196^ ^]^ Although polymeric nanocarriers not only increase their stability, reduce side effects, deliver drugs specifically to tumor tissues via the EPR effect, the gap between the inefficient drug release rate in tumor and sufficient intracellular drug concentration request for cancer therapy remained. Driven the host‐guest interaction water‐soluble pillar[5]arene and a boronate ester linked CUR formed nanoprodrug^[197]^ with dual‐responsiveness toward pH and GSH (Figure 13E), allowing the selective release of drug in hepatocellualr cells. It is believed that the PEGylation of nanoassemblies significantly prolonged the circulation time of PTX, Sun et al. therefore conjugated PEG 2000 to PPa then PEGylated PTX‐S‐OA prodrug into NPs which fulfilled synergistic chemophotodynamic therapy under laser irradiation, attributing to the single oxygen (^1^O_2_) generated by PPa not only raised PDT but promoted PTX release in response to the overproduced ROS in tumour cells.^[^ ^198^ ^]^ More sophisticated multiple stimuli‐activated tumor‐specific dendrimer nanoassemblies was developed to sequentially overcome physiological barriers of drug resistance (Figure 13F), including tumor microenvironment enzyme‐driven prodrug NPs transportation to deepen tumor penetration and cellular uptake, lysosome acid‐triggered nucleus delivery of antitumor drugs and cytoplasmic redox‐sensitive breakdown for sufficient release of encapsulated agents.^[^ ^199^ ^]^ Not only small chemodrugs, but biomacromolecules can be incorporated into the polymeric nanocarriers. For instance, ribonuclease A was incorporated into the triblock copolymer NPs via both ROS‐cleavable carbamate bond and pH‐reversible phenylboronic acid‐catechol linkage, together with encapsulated DOX fulfilled enhanced synergistic anticancer effects.^[^ ^200^ ^]^

## 4 Biomacromolecule‐Drug Conjugates

Naturally‐occurred monomeric species, such as amino acids, nucleic acids and monosaccharides linked together through peptide, glycosidic and phosphodiester bonds into biopolymers, representing by the proteins, DNA, polysaccharide (PS).

### 4.1 Polysaccharide‐Drug Conjugates

**Figure 14:**
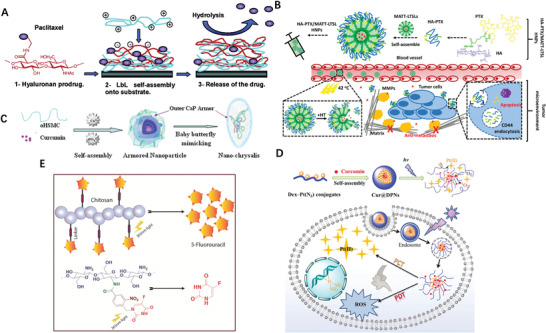
A) Scheme of hyaluronan‐PTX prodrugs multilayers construction through layer‐by‐layer approach and drug release mechanism. Reproduced with permission.^[^ ^201^ ^]^ Copyright 2005, American Chemical Society. B) Schematic illustration of HNP preparation and intended mechanism for metastatic breast cancer treatment. Reproduced with permission.^[^ ^204^ ^]^ Copyright 2018, American Chemical Society. C) A schematic illustration of the procedure for preparing nano‐chrysalis. Reproduced with permission.^[^ ^206^ ^]^ Copyright 2017, Elsevier. D) Preparation and single‐stimulus (Light) dual‐drug sensitivity of Cur@DPNs and schematic representation of the intracellular action after endocytosis of Cur@DPNs for combinational photoactivated therapy. Reproduced with permission.^[^ ^209^ ^]^ Copyright 2016, American Chemical Society. E) Release mechanism of 5‐Fluorouracil from the LMWC‐5 FU conjugate prodrug upon irradiating with *λ*  =  365 nm light. Reproduced with permission.^[^ ^217^ ^]^ Copyright 2019, Elsevier.

PS is low toxic, biocompatible, biodegradable, stable, hydrophilic, bioadhesive, and chemical‐modification facile and has been extensively investigated as building blocks to construct drug delivery system. The typical arrangement is a drug linked to a biocompatible polymer via hydrolyzable linkages. This methodology represents remarkable advantages to solubilize hydrophobic drugs and tune the drug pharmacokinetics. Thierry and co‐workers utilized a hyaluronan (HA)‐prodrug of PTX with liable succinate ester bond to construct the polyelectrolyte multilayers (PEMs) with polyamine CS (**Figure** **14**A); the obtained PEMs (PTX loading of 3% versus HA disaccharide units) was soluble in NaCl solution displayed a 95% reduction of murine macrophages J774.^[^ ^201^ ^]^ Through click reaction, DOX was grafted to HA and obtained core–shell NPs.^[^ ^202^ ^]^ HA, a water soluble biocompatible and biodegradable polymer, can function as a targeting agent and delivery vehicle in many DDS. HA specifically recognize various cancer cells that overexpressed HA receptors such as CD44 on the cell surface. The nanosized HA particles, consisted of a hydrophobic anticancer adamplatin prodrug core and hydrophilic CD/HA shell, showed comparable anticancer activities to the commercial drug cisplatin but remarkably reduced toxicity in vitro and in vivo.^[^ ^203^ ^]^ HA‐PTX prodrug self‐assembled on the surface of marimastat‐loaded thermosensitive liposomes to form spherical hybrid drug‐loaded NPs; triggered by mild hyperthermia, the NPs rapidly released HA‐PTX and entered 4T1 cells due to CD44‐HA high affinity (Figure 14B).^[^ ^204^ ^]^ The semisynthetic hydroxyethyl starch (HES)‐DOX prodrug conjugates through disulfide bond with diameter of ≈20 nm fulfilled tumor targeted drug delivery and GSH‐mediated intracellular release.^[^ ^205^ ^]^ A hybrid tumor microenvironment‐mediated dual pH/redox‐responsive polymeric NPs combined with calcium phosphate (CaP) was reported, which has an inner core composed of oligosaccharides of HA‐fluorescent CUR‐prodrug and outer armor of CaP (Figure 14C).^[^ ^206^ ^]^ The *α*‐Amylase can degrade the HES shell of HES‐SS‐PTX prodrug NPs, smoothing NP extravasation and penetrating into the tumor, consequently showed improved 11.2% in vivo antitumor efficacy in 4T1 tumor‐bearing mice compared with those of Taxol.^[^ ^207^ ^]^ Heparin is a biofriendly, water‐soluble natural PSs and rich in animal tissues. The conjugation of PTX with heparin through single amino acid spacers self‐assembled into NPs in aqueous while maintaining structural integrity; besides the improved properties in vitro and in vivo, the obtained NPs reducing the risk of severe hemorrhagic complication during administration.^[^ ^208^ ^]^

Another commonly used PS is dextran (Dex), He et al. grafted hydrophobic prodrug Pt(N_3_) to Dex for CUR encapsulation and self‐assembling of NPs which were believed to have dual modes of action upon cancer cells: CUR would be photoactivated to generate instant ROS within cancer cell for fast PDT, and simultaneously photoreduced Pt(N_3_) into active Pt(II) for long‐acting PCT (Figure 14D).^[^ ^209^ ^]^ Both hydrophobic CPT and hydrophilic polymer were grafted with Dex to get self‐assembled amphiphilic prodrug in aqueous.^[^ ^210^ ^]^ Cinnamaldehyde‐conjugated maltodextrin (CMD) was developed as a polymeric prodrug of cinnamaldehyde and a drug nanocarrier for CPT.^[^ ^211^ ^]^

The nanoprodrug strategy based on chitosan (CS) for efficient intracellular DOX delivery was reported in several cases.^[^ ^212^, ^213^, ^214^ ^]^ Strong oxidization led to the CS ring open and the exposed aldehyde moiety can react with the imine group of metformin to offer grafted prodrug which can formed nanocomplexes with therapeutic gene through electrostatic interactions.^[^ ^215^ ^]^ Folic acid and was decorated on the surface of CS prodrug NPs for improved intracellular accumulation of 6‐mercaptopurine (6‐MP) in leukemia.^[^ ^216^ ^]^ The amine terminal of CS is readily to react with carboxylic acid group, hence Horo et conjugated 5‐fluorouracil(5‐FU) to CS through photocleavable linker containing *o*‐nitrobezyl analogue, then the conjugate prodrug was treated with sodium tripolyphosphate and formed into controlled released NPs upon irradiating with *λ* = 365 nm light (Figure 14E).^[^ ^217^ ^]^ To improve the loading efficacy of hydrophilic 5‐FU, its lipophilic prodrug 5‐FU‐stearic acid was synthesized and encapsulated into the core of xylan‐SA NPs through hydrophobic interaction.^[^ ^218^ ^]^

### 4.2 Polypeptide‐Drug Conjugates

Beyond PS, few types of biomacromolecule have been investigated in the context of self‐assembled prodrug nanomedicine. Covalent join of a drug with a peptide moiety into a prodrug is an effective method to ameliorate the drug's therapeutic outcome. The cell membrane penetrating peptide Tat is covalently modified by different number of DOX; it turned out that cellular accumulation efficiency and the free DOX release rate are determinant to the in vitro efficacy of drug conjugates.^[^ ^219^ ^]^ The peptide sometime could be dually functional, meaning not only the linker of the components, but help to hold second therapeutics. For instance the chelation of GFFYERGD with cisplatin enable the formation of nanohydrogel of Rh‐peptide conjugates.^[^ ^220^ ^]^ Targeted delivery with peptide involvement as effective prodrug approaches has been comprehensively reviewed by Cui et al. few years before.^[^ ^221^ ^]^ Polypeptides, being biocompatible and biodegradable, have been commonly utilized as nanoengineered carriers for drug delivery.

**Figure 15:**
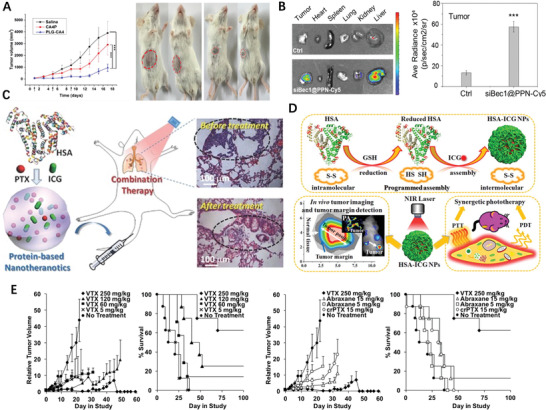
A) C26 tumor volumes after injection with saline, PLG‐CA4 and CA4P on days 1, 5, and 9; Images of C26 tumors on day 11, mice on the left were treated with CA4P and mice on the right were treated with PLG‐CA4. Reproduced with permission.^[^ ^225^ ^]^ Copyright 2017, Elsevier. B) siBec1@PPN effectively suppressed Pt‐resistant tumor growth. Reproduced with permission.^[^ ^228^ ^]^ Copyright 2019, American Chemical Society. C) A schematic illustration to show the formation of HAS‐ICG‐PTX NPs by self‐assembly between HSA, PTX, and ICG. Reproduced with permission.^[^ ^235^ ^]^ Copyright 2015, Wiley‐VCH. D) Schematic illustration of the programmed assembly strategy for the preparation of HSA‐ICG NPs and synergetic phototherapy. Reproduced with permission.^[^ ^236^ ^]^ Copyright 2014, American Chemical Society. E) Efficacy and survival in HT‐1080 fibrosarcoma model (*n* = 8 per group). Reproduced with permission.^[^ ^237^ ^]^ Copyright 2019, American Chemical Society.

Receptor‐adjusted endocytosis is the dominant mechanism for targeted drug delivery to cancer tissues, which is certainly suitable for prodrug case. To bypass the blood brain tumor barrier, Jiang et al. functionalized PTX prodrug self‐assembled NPs (PSNPs) with glioma homing peptide Pep‐1; Pep‐PSNPs could significantly enhance cellular uptake in U87MG cells comparing to peptide free PEG‐PSNPs.^[^ ^222^ ^]^ Targeting peptide‐drug prodrug was also loaded inside of amphiphilic copolymer coated hollow mesoporous copper sulfide hybrid nanosystem.^[^ ^223^ ^]^ By mixing negatively charged poly(L‐glutamic acid), Pt prodrug, carboxyl‐modified Fe_3_O_4_ NPs and positively charged poly(L‐lysine) through electrostatic interaction, Gao et al. constructed a polypeptide‐based nano‐vehicle which fulfilled the combined antitumor treatment by released Pt drug and generated sufficient ^•^OH, meanwhile used for *T*_2_‐weighted MRI of tumor.^[^ ^224^ ^]^ CA4 represents a critical vascular disrupting strategy for tumor therapy, but the anti‐polymerization effect of CA4 on tubulin is reversible and its rapid biological clearance has compromised its overall therapeutic efficiency. To fully potentialize CA4, Liu et al. proposed a poly(l‐glutamic acid)‐CA4 conjugate(PLG‐CA4) prodrug nanomedicine which resulted in a 74% tumor suppression rate while commercial CA4 phosphate only showed 24%, attributing to the PLG‐CA4 being largely spread around the tumor vessels (**Figure** **15**A).^[^ ^225^ ^]^

Chen constructed a nanoprodrug in which DOX was shielded in the core by a folate acid (FA)‐modified proapoptotic peptide (KLAKLAK)_2_ to antineoplastic agent.^[^ ^226^ ^]^ Chilkotia et al. conjugated silica promoting elastin‐like polypeptide and DOX that self‐assemble into micelles with silica shell encapsulated yet underwent the programmable release of drugs in response to pH shift.^[^ ^227^ ^]^ To cure cisplatin‐resistant lung cancer, Lin developed a self‐assembled nanoprodrug platform consisting of three types of modules where Pt(IV) was fixed with cationic peptide, allowing the Pt(IV) a loading efficiency of >95%; the peptide of the prodrug complex efficiently deliver Beclin1 siRNA and accumulate into tumor (Figure 15B), therefore synergistically inhibited the growth of a drug‐resistant tumor on xenograft mice bioassy up to 84% after intravenous injection.^[^ ^228^ ^]^

Naturally occurred polypeptide, on the other hand, outperform the synthetically derived ones beyond providing solubility and avoiding fast clearance in vivo. Albumin, produced by the liver that circulates in plasma, is the most abundant and perhaps most important protein in human blood. It is an attractive drug delivery platform;^[^ ^229^, ^230^ ^]^ Noticeably, albumin has high affinity with tumors and sites of inflammation, the feature which pave the way for targeting functionality.^[^ ^231^ ^]^ Protein‐bound PTX with trade name Abraxane, also known as NP albumin‐bound PTX or nab‐PTX. This suspension formulation, utilized the natural properties of albumin to reversibly bind PTX, transport across the endothelial cell and accumulate PTX in tumor tissues,^[^ ^232^ ^]^ was be believed a successful application of prodrug‐nanotechnology. However, Abraxane still cause remarkable side effects when dose high.^[^ ^233^ ^]^ Therefore, more attempts based on PTX formulation are still urgently needed. A “Abraxane‐like” prodrug formulation, comprised of human serum albumin (HSA), a dimeric PTX joined with thioether liner (PTX_2_‐S), and photosensitizer was reported that dramatically increase drug loading content from 6.6 to 48.7 wt% comparing to Abraxane itself.^[^ ^234^ ^]^ Mechanical mixing of HSA, PTX, and indocyanine green (ICG) in the aqueous solution gave self‐assemble into NPs (Figure 15C) which was more stable and circulated longer in blood compared with HSA‐ICG complex.^[^ ^235^ ^]^ This upgraded version of “Abraxane” also enabled the mild photothermal heating under the NIR laser irradiation and promoted the intracellular uptake of HAS‐ICG‐PTX for improved cancer cell killing.^[^ ^235^ ^]^ HSA was unfolded with excessive GSH, then loaded ICG to form HSA‐ICG NPs through intermolecular disulfide conjugations under mild oxidization condition (Figure 15D); the HSA‐ICG NPs showed a high accumulation with tumor‐to‐normal tissue ratio and a long‐term retention with more than one week in tumor‐bearing mice. Besides, HSA‐ICG NPs efficiently simultaneously induced ROS and local hyperthermia, therefore achieved synergetic PDT/PTT treatments under a single NIR laser irradiation.^[^ ^236^ ^]^ The continued efforts to improve the PTX‐protein formulation lead to the discovery of long‐chain fatty acids (LCFAs)‐PTX prodrug that can readily formed noncovalent complex with HAS, kept LCFAs binding pose unchanged, differed in pharmacokinetics, shown higher maximum tolerated doses (Figure 15E) and increased efficacy in vivo in multiple cancer models, compared to clinical formulations Abraxane and Cremophor EL‐formulated PTX.^[^ ^237^ ^]^

### 4.3 Nucleic Acid‐Drug Conjugates

**Figure 16:**
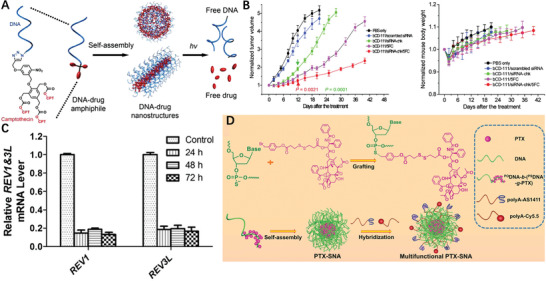
A) Schematics of the DNA–Drug Nanostructures Assembled from Photolabile DNA‐Drug Amphiphiles. Reproduced with permission.^[^ ^240^ ^]^ Copyright 2015, American Chemical Society. B) Growth curves of tumors treated with a dose of nanoplex; Reproduced with permission.^[^ ^242^ ^]^ Copyright 2010, American Chemical Society. C) qRT‐PCR confirmation of REV1 and REV3L gene suppression in LNCaP cells that were harvested from a xenograft tumor and isolated by GFP sorting 2 or 3 d after injection of NP (siREV1, siREV3L). Reproduced with permission.^[^ ^243^ ^]^ Copyright 2013, The National Academy of Sciences. D) Schematics of DNA‐graft‐PTX conjugate synthesis, and the self‐assembly of multifunctional PTX‐SNAs. Reproduced with permission.^[^ ^246^ ^]^ Copyright 2019, Wiley‐VCH.

Recently, controlled arrangements of nucleic acids into specific nanoarchitectures can assign unusual properties and found themselves braoder biomedical applications,^[^ ^238^ ^]^ drug delivery in particular.^[^ ^239^ ^]^ It is thought that the co‐delivery of nucleic acids and small chemodrugs allow readily access to gene and protein targets; spherical nucleic acids (SNAs) rapid endocytosis makes them suitable for drug delivery to overcome anticancer drug resistance. Tan et al. reported a self‐delivery form of nucleic acid‐CPT prodrug nanostructure which sheds the nucleic acid shell (**Figure** **16**A) upon light activation, disassembled via an irreversible self‐immolative process to liberate free CPT.^[^ ^240^ ^]^ Cocktail therapy of combination drug for cancer treatment may ineffective because of pharmacokinetics diversity of different drugs. Many drug delivery systems can transport multidrugs, but few sustain a persistent and precise drug release ratio because of the chemophysical properties difference of individual drugs. To address these challenges, Zhou et al. developed a circular bivalent aptamer‐CPT conjugates with tunable drug ratios for an enhanced synergistic effect.^[^ ^241^ ^]^ A nanoplex containing magnetic resonance imaging reporters, siRNA and enzymatic therapy resulted in 6‐folds increase of tumor doubling time comparing to reference (Figure 16B), in which Gd^3+^‐DOTA for in vivo detection, FITC for microscopy to image, and a prodrug enzyme bacterial cytosine deaminase that converts the nontoxic to cytotoxic 5‐fluorouracil in breast tumors.^[^ ^242^ ^]^

The ideal cancer treatments strategy target to cancer cells while minimize normal tissue damage. Among them, siRNA‐mediated silencing of specific targets is commonly used to achieve cancer cure. To this end, Xu et al. developed a NP platform to deliver a cisplatin prodrug and *REV1/REV3L*‐specific siRNAs simultaneously, PCR results showed that the NPs exhibited a significant and sustained suppression of tumor genes in for up to 3 days (Figure 16C) after a single dose.^[^ ^243^ ^]^ The conjugation of tumor‐targeting aptamer AS1411 with PTX not only improve the solubility of drug but enable ovarian cancer targeting.^[^ ^244^ ^]^ Amphiphilic DNA‐PTX conjugate is synthesized by conjugating antisense oligonucleotide with PTX through reduction‐activatable linkage, then further self‐assembled into PTX‐cored spherical nucleic acids (SNAs); the SNAs enter cells ≈ 100 times faster than free DNA, showed nearly identical cytotoxicity as free PTX.^[^ ^245^ ^]^ A more comprehensive drug delivery SNAs‐like micellar NPs hybridizing nucleic acid‐PTX, aptamer AS1411 and fluorescent probe cy5.5 (Figure 16D), fulfills not only up to ≈53% drug loading but provides the targeting and imaging functionalities.^[^ ^246^ ^]^ Cisplatin prodrug and DOX were incorporated with DNA duplex‐modified gold nanorods through covalent and non‐covalent individually then realized co‐delivery.^[^ ^247^ ^]^

## 5 Remark and Outlook

The development of prodrugs satisfies the desire of the patients who expect more efficacious treatment with mitigated toxicity compared to standard drug formulations and match the motivation of big pharma whose preference is to invest on the cost‐efficient project. Drugs with tolerant side effects attract in particular the attentions of medicinal chemists since it is that these imperfect ones serve as a decent starting point for further modifications/improvements since *de novo* design is more time‐consuming and challenging. Recently, some unusual prodrugs have been newly developed, such as organoarsenic^[^ ^248^ ^]^ and antimony prodrugs,^[^ ^249^ ^]^ which enriched our arsenal to combat with hazardous diseases, including malignant cancer.

Nanomedicine, fuled by nanotechnology, manipulates therapeutical materials in nanoscale and promises scientific even clinic advancement in disease treatment. Nanoparticulation can improve water solubility and stability of medicine with/without using extra excipients. Physical encapsulation using nanocarriers and chemical conjugation through covalent bonds are two major scenario for nanomedicine formulation and both of them remain popularized.

The prodrugs transformation is a common approach to confront drug delivery challenges by assigning parent drug “warhead” or “prosthesis”, nanotechnology is a powerful tool to enhance drug delivery for rationalizing the biodistribution of the encapsulated drug payloads. Nanoformulation of prodrugs, a combination of prodrug strategy and nanotechnology tactics, inherits the merits both hence holds a few advantages, such as high drug loading efficiency, improved drug availability and enhanced accumulation in cancer cells in terms of drug delivery. Reactive drug loading, decent stability, and triggered drug release and targeted therapy^[^ ^250^ ^]^ are the four essential components of a smart nanoprodrug platform and should improve the pharmacokinetics and decrease toxicity of the chemotherapeutic by transforming an encapsulated prodrug to tumor sites, where it is selectively released and converted to active parent drug.

Unlike conventional drug delivery platform based on polymer with drug physically trapped in, self‐assembled polymeric prodrug delivery vehicle often covalently tether drug therefore is more stable and could avoid premature release, on the other hand spatiotemporally controllable release requires stimuli‐responsive linkers. Polymeric nanoscale DDS can increase the solubility of parent drug as well as enable passive targeting to the solid tumor. It is believed that cancer cells differ from normal ones primarily being the cancer cells divide uncontrollably while normal cells stop growing (reproducing) when enough cells are present. Except the polymer mediated nanoprodrug delivery strategy, prodrug self‐delivery systems with nanoscale characteristic realize intracellular delivery by themselves without the aid of excipient have shown multiple advantages, such as high drug loading, convinced molecule weight, controllable drug ratio, enhanced stability and synergetic outcome. The cancerous tissue is rich of acidic, reducing and ROS ingredients, overexpressed receptors in tumor microenvironment and vulnerable immune system comparing to normal ones, they are all specifically available stimulus to trigger drug release; it is believed that these features together with the EPR effects are the fundamental of nanoprodrug for targeting cancer treatments. It is important to emphasize that the linker/spacer between drug and macromolecular/drug need to be chemically or enzymatically hydrozable to liberate original drug. Therefore, the liability of prodrug linker is critical for macromolecular carrier/drug‐spacer‐drug conjugate system therefore require thoughtful design. Luckily, the abovementioned features exclusive presenting in cancer cells can be utilized for stimuli‐responsive linker selection/screening. The linker between prodrug components needs to be stimuli‐sensitive enough since unbreakable or slowly hydrolyzed bond will deteriorate the therapeutical effect of parent drugs. One should keep in mind several key aspects when the prodrug components are covalently connected: is the linker really needed? How long of the linker should be? To which stimuli the linker should respond? Is the prodrug linker extracellularly stable yet intracellularly labile for drug retention during blood circulation but fast drug release within the cell? A nanoprodrug could undergo different sizes or surface charges change in different delivery stages for optimal performance is desired, which is achievable by incorporating tumor‐acidity‐cleavable maleic acid amide^[^ ^251^ ^]^ linker for instance. Beside, ones may keep eyes on novel drug liberation mechanism such as mechanochemistry.^[^ ^252^ ^]^

The detailed mechanism of nanoprodrug assembling remains in its infancy therefore deserve more investigation, in particular the behavior of NPs at fluid‐fluid interface.^[^ ^253^ ^]^ The in situ observation of NPs formation, growth process and trajectory tracking are often technologically challenged and expensive. Thanks to the ever‐growing computational hardware and technology,^[^ ^56^ ^]^ it is promising these insights could be decoded in foreseeable future using *in silicon* techniques. Pharmaceutical formulation developments, including most assembling of nanoprodrug is complicated and idea formulations are generally attained after extensive wet lab procedure. Computational, particularly machine learning and molecular dynamics simulations,^[^ ^254^, ^255^ ^]^ may provide molecular understanding of prodrug behavior that help rationalized manipulation of self‐assembled structures before start the actual preparation, standardize/streamline the formulation development while currently the process highly depends on the trial‐and‐error experiences of individual formulation practitioners. The integration of computational tools into nanoprodrug design would significantly accelerate the successful rate and practically aimful to screen the desired nanomedicines. For instance based on docking results, the optimal laurate functionalized Pt(IV)‐prodrugs was designed and synthesized since its complex with HAS possessed the lowest binding energy, therefore the obtained NPs might be stable enough to maintain their structures during sonication was applied and would lead to Pt release from HAS intracellularly.^[^ ^256^ ^]^ Of course we should not overemphasize the role of computational simulation in drug formulation process. Similar to the application to other research field, a useful computational pharmaceutics results should importantly keep agreement with experimental outcomes. For example, pharmacokinetics simulation of the micelle, serum and tumor compartments in a three compartmental model implied that improved tumor drug delivery was related to the decreased rates of drug release from the micelles to the serum, which successfully interpreted the observation of co‐delivery PTX and alkylated cisplatin prodrug in polymeric micelles.^[^ ^257^ ^]^

Similar to other type of nanomedicines, the size of micellar nanoprodrug also plays a key role for their fate in body. Both too large and too small micelle in size deteriorate the function of prodrug nanomedicinesIn principle, larger vesicles circulated in blood longer and accumulated more in tumor, however the massive tumor accumulation of the large micelles does not mean improvement of therapeutic efficacy since the large micelle poorly penetrate in deeper tumor tissues than the small ones. Therefore, an optimal size that balances drug accumulation and penetration in tumors should be pursued.^[^ ^258^ ^]^ Although the size and morphology of nanoprodrug are the prodrugs complex themselves determined indeed, these features can be tuned in certain degree by using some technology, such as microfluidics to fabricated quality controlled products^[^ ^259^ ^]^ with high productivity hence potentialize clinical translation.

Although research in nanoprodrug has been growing rapidly in recent years, and many of show eye‐catching results preclinical studies, unfortunately only few of them show clinical efficacy advantages. The discrepancy obtained could be attributable to species differences and limitations of animal disease models. Firstly, drug release from prodrug NPs and its pharmacokinetic profile in mice could be very different than that in humans because mice have different enzyme and/or transporter activities.^[^ ^260^ ^]^ Thus, drug release and dosing regimen for animal must be reevaluated for humans. Secondly, for cancer research, animal disease models might not fully recapitulate human malignancies because of the differences in tumor size/body weight, tumor growth rate, and tumor microenvironment. For example, a xenografted tumor in a nude mouse is ≈0.3–30% of mouse weight whereas a tumor in a human patient is only 0.003–0.01% of body weight. Because of the much bigger size of implanted tumors in mice, it takes only six seconds for NPs to reach the tumors in mice, but over ten days in human patients.^[^ ^261^ ^]^ Moreover, in many mouse studies, implanted tumors are growing rapidly accompanied by angiogenesis, which makes the EPR effect more pronounced.^[^ ^262^ ^]^ Although the EPR effect has been the main principle for the design of drug NPs to date, its relevance to human tumors remains to be investigated. Most preclinical studies have used either mice bearing mouse cancer cells or athymic nude mice bearing human cancer cells. They all have limitations in recapitulating human diseases. Responses of mouse cancer cells to drug NPs hardly translate into responses of human cancer cells in clinical trials. Athymic nude mice have T cell deficiency and thus have different tumor immunoenvironment. Tumor‐infiltrating immune cells are part of tumor microenvironment and determinant in tumor growth.^[^ ^263^ ^]^ Although the exploitation of nanoprodrug for cancer treatments is far beyond the satisfaction or on‐demand trend, it brighten a new avenue and is superior to classical drug delivery system from several facets.

## Conflict of Interest

The authors declare no conflict of interest.
